# Trends in Incidence of Intracerebral Hemorrhage and Association With Antithrombotic Drug Use in Denmark, 2005-2018

**DOI:** 10.1001/jamanetworkopen.2021.8380

**Published:** 2021-05-05

**Authors:** Stine Munk Hald, Sören Möller, Luis Alberto García Rodríguez, Rustam Al-Shahi Salman, Mike Sharma, Hanne Christensen, Maja Hellfritzsch, Anton Pottegård, Jesper Hallas, David Gaist

**Affiliations:** 1 Research Unit for Neurology, Odense University Hospital, Odense, Denmark; 2 University of Southern Denmark, Odense; 3 Open Patient Data Explorative Network (OPEN), Odense University Hospital, Odense, Denmark; 4 Department of Clinical Research, University of Southern Denmark, Odense; 5 Centro Español Investigación Farmacoepidemiológica, Madrid, Spain; 6 Centre for Clinical Brain Sciences, University of Edinburgh, Edinburgh, United Kingdom; 7 Division of Neurology, Department of Medicine, Population Health Research Institute, McMaster University, Hamilton, Ontario, Canada; 8 Department of Neurology, Bispebjerg Hospital and University of Copenhagen, Copenhagen, Denmark; 9 Department of Clinical Pharmacology, Aarhus University Hospital, Aarhus, Denmark; 10 Department of Clinical Pharmacology and Pharmacy, University of Southern Denmark, Odense

## Abstract

**Importance:**

Spontaneous (nontraumatic) intracerebral hemorrhage (ICH) is the most severe complication of antithrombotic drug use.

**Objectives:**

To estimate the strength of association between use of antithrombotic drugs and risk of ICH and to examine major changes in the incidence of ICH in the general population.

**Design, Setting, and Participants:**

This case-control study of patients with a first-ever ICH from January 1, 2005, to December 31, 2018, matched by age, sex, and calendar year with general population controls (1:40 ratio), assessed case and control patients 20 to 99 years of age in population-based nationwide registries in Denmark (population of 5.8 million).

**Exposures:**

Use of low-dose aspirin, clopidogrel, a vitamin K antagonist (VKA), or a direct oral anticoagulant (DOAC).

**Main Outcomes and Measures:**

Association of ICH with antithrombotic drug use, annual age- and sex-standardized incidence rate of ICH, and prevalence of treatment with antithrombotic drugs. Conditional logistic regression models estimated adjusted odds ratios (aORs) (95% CIs) for the association of antithrombotic drugs with ICH.

**Results:**

Among 16 765 cases with ICH (mean [SD] age, 72.8 [13.1] years; 8761 [52.3%] male), 7473 (44.6%) were exposed to antithrombotic medications at the time of ICH onset. The association with ICH was weakest for current use of low-dose aspirin (cases: 28.7%, controls: 22.6%; aOR, 1.51; 95% CI, 1.44-1.59) and clopidogrel (cases: 6.2%, controls: 3.4%; aOR, 1.65; 95% CI, 1.47-1.84) and strongest with current use of a VKA (cases: 12.0%, controls: 5.0%; aOR, 2.76; 95% CI, 2.58-2.96). The association with ICH was weaker for DOACs (cases: 3.0%, controls: 1.8%; aOR, 1.83; 95% CI, 1.61-2.07) than for VKAs. Compared with 2005, the prevalence of use of oral anticoagulants among general population controls in 2018 was higher (3.8% vs 11.1%), predominantly because of increased use of DOACs (DOACs: 0% vs 7.0%; VKA: 3.8% vs 4.2%). Antiplatelet drugs were used less frequently (24.7% vs 21.4%) because of decreased use of low-dose aspirin (24.3% vs 15.3%), whereas clopidogrel use increased (1.0% vs 6.8%). The age- and sex-standardized incidence rate of ICH decreased from 33 per 100 000 person-years in 2005 to 24 per 100 000 person-years in 2018 (*P* < .001 for trend).

**Conclusions and Relevance:**

In Denmark from 2005 to 2018, use of antithrombotic drugs, especially VKAs, was associated with ICH. Although use of oral anticoagulation increased substantially during the study period, the incidence rate of ICH decreased.

## Key Points

**Question:**

Is use of antithrombotics associated with intracerebral hemorrhage (ICH), and is increased use of these drugs in recent years associated with increasing incidence of ICH in Denmark?

**Findings:**

In this case-control study of 16 765 ICH cases, ICH was statistically significantly associated with antithrombotic drug use, including vitamin K antagonists, direct oral anticoagulants, clopidogrel, and low-dose aspirin. Whereas antithrombotic drug use (mainly direct oral anticoagulants) increased during 2005 to 2018, the incidence of ICH did not.

**Meaning:**

In Denmark, increased use of antithrombotics was not associated with an increase in the incidence of ICH.

## Teaser

This case-control study estimates the association between use of antithrombotic drugs and risk of intracerebral hemorrhage and assesses changes in the incidence of intracerebral hemorrhage in the general population in Denmark.

## Introduction

Anticoagulant and antiplatelet drugs offer clear clinical benefits in the treatment and prevention of thrombosis, but their use is also associated with an increased risk of intracerebral hemorrhage (ICH).^1,2,3^ Although less common than gastrointestinal hemorrhage, ICH is a severe complication with high case fatality in the setting of antithrombotic therapy.^3,4^ Therefore, the advent of direct oral anticoagulants (DOACs) that were reported to be associated with reduced risk of ICH compared with warfarin in clinical trials in patients with atrial fibrillation^3^ represented a major advance in oral anticoagulant (OAC) therapy. However, although intracranial hemorrhages (encompassing intracerebral, subdural, and subarachnoid hemorrhages) have been extensively studied as untoward effects of DOACs in observational studies,^2,5,6,7,8,9,10,11,12,13,14,15,16,17,18,19,20,21,22,23,24,25,26,27,28^ this is less the case for ICH. Observational studies that specifically present data on the risk of ICH associated with OAC use mostly predate the introduction^1,29,30^ and more widespread use of DOACs^2^ or report data only on patients with atrial fibrillation.^31^ Currently, preadmission use of OACs—alone or combined with antiplatelets—is relatively frequent among patients admitted for ICH.^32^ Antithrombotic drugs are frequently used by older people, the demographic segment with the largest projected increase worldwide in years to come.^33^ Observational studies can provide insights regarding the association of ICH with antithrombotic drug use in the wider population, including vulnerable populations, such as older people and patients with coexisting conditions, who are often less well represented in clinical trials.

Therefore, we conducted a large, population-based study to provide detailed analyses of the association of use of OACs and antiplatelet drugs with risk of ICH. Furthermore, we examined whether major changes in the landscape of antithrombotic therapy^34,35,36^ in recent years have impacted the incidence rate of ICH,^37^ as reported for warfarin in the pre-DOAC era.^38,39^

## Methods

In Denmark (population of 5.8 million), health services, including hospital care, are tax financed and free of charge for all residents. All services are registered in nationwide registries under the same unique person identifier, allowing complete linkage among registries. Within this setting, we performed case-control analyses and descriptive analyses. In accordance with Danish law regarding register-based research, the study was approved by the Region of Southern Denmark, and informed consent was waived.^40^ Data were pseudonymized. This study followed the Strengthening the Reporting of Observational Studies in Epidemiology (STROBE) reporting guideline.

### Source Population for Case-Control Study

The source population, providing cases and controls for this study, was all people 20 to 99 years of age followed up from January 1, 2005, to December 31, 2018. We excluded people from the source population if they had been residents of Denmark for less than 10 years or if they had a prior diagnosis of ICH.

### Cases and Controls

We defined cases as people within the source population who were admitted in 2005 to 2018 with a first-ever diagnosis of spontaneous (nontraumatic) ICH according to the Danish Stroke Registry (Stroke Registry).^41^ The date of admission was used as the index date. We sampled the Danish Civil Registration System^42^ to identify 40 controls without ICH from the source population for each case. Using risk-set sampling, we matched controls by birth year and sex to their index case and assigned an index date identical to the index date of their corresponding case.^43^

### Exposure to Antithrombotic Drugs

We determined exposure to antithrombotic drugs for cases and controls based on prescriptions dispensed from 1995 and up to 1 day before the index date according to information from the Danish National Prescription Registry (Prescription Registry).^44^ In Denmark, low-dose aspirin is the only antithrombotic available over the counter. In the study period, 90% or more of low-dose aspirin was dispensed per prescription and therefore recorded in the Prescription Registry.^45^ On the basis of the most recent episode of antithrombotic drug use, we classified this exposure into the following categories: current use (prescription supply ended 0-30 days before index date), recent use (31-90 days before index date), past use (91-365 days before the index date), and nonuse (no supply in 365 days before the index date). We classified prescribed antiplatelet drugs into low-dose aspirin (only available in doses of ≤150 mg in Denmark), clopidogrel, prasugrel, ticagrelor, or dipyridamole and OACs into vitamin K antagonists (VKAs; warfarin and phenprocoumon) and DOACs (dabigatran, rivaroxaban, apixaban, and edoxaban). Drug exposure assessment is further detailed in the eMethods in the Supplement.

### Potential Confounders

We used data from the Danish National Patient Register^46^ (Patient Registry) and the Prescription Registry to classify each individual’s history of disorders and use of concomitant medications that we regarded as potential confounders (listed below; for codes, see eTable 1 in the Supplement).

### Statistical Analysis

We used conditional logistic regression to compute adjusted odds ratios (aORs) and 95% CIs for ICH associated with use of low-dose aspirin, clopidogrel, VKAs (as a class and for warfarin), and DOACs (as a class and for individual drugs) vs nonuse of antithrombotic drugs. The effect of dipyridamole alone, with no concurrent use of low-dose aspirin, was not analyzed because, in accordance with Danish stroke prevention guidelines, dipyridamole is only recommended in combination with low-dose aspirin.^47^ Because use of prasugrel and ticagrelor was limited during the study period, we did not perform analyses restricted to these drugs.

We adjusted analyses for the following potential confounders: history of chronic obstructive pulmonary disease (as a marker of smoking); disorders indicative of high alcohol consumption; hypertension; ischemic stroke; diabetes; chronic hepatic diseases; chronic renal failure; heart failure; ischemic heart disease; peripheral artery disease; cancer; coagulopathy; and current use of nonsteroidal anti-inflammatory drugs, selective serotonin reuptake inhibitors, statins, hormone replacement therapy, or oral corticosteroid drugs. In analyses that focused on single antiplatelet drug use, we also adjusted for current use of OACs. Similarly, in analyses for single OACs, we adjusted for current use of antiplatelet drugs. We chose covariates included in the model described above, based on current subject matter knowledge regarding potential confounders and known risk factors for ICH. We evaluated differences in strength of association between subgroups or between different outcomes by using the 2-sample Wald test.

In descriptive analyses, we estimated the annual incidence rate (IR) of ICH and fatal ICH (eMethods in the Supplement) and corresponding age- and sex-standardized IRs using census data from Statistics Denmark. We tested for trend (Poisson regression; eMethods in the Supplement) in annual standardized IRs in the entire study period. For 4 age groups (20-64, 65-74, 75-84, and ≥85 years), we calculated the incidence rate ratios (IRRs) for ICH in the second half of the study period (2012-2018) using the first half of the study period (2005-2011) as reference. For the same 2 periods, we calculated prevalence ratios of current use of antithrombotics in the general population, as represented by the exposure history of the controls.

We conducted several supplementary analyses, including analyses stratified by age and sex, recency and duration of antithrombotic drug use, indication for use (atrial fibrillation vs venous thromboembolism), and new use and naive use analyses (eMethods in the Supplement). Finally, we gathered medical record information on patients recorded with an ICH diagnosis (Stroke Registry or Patient Registry) in the Region of Southern Denmark (population of 1.2 million) in 2009-2017.^48^ In this regional subset, we calculated standardized IRs and IRRs of verified spontaneous ICH for 3-year time-bands (eMethods in the Supplement).

Two-tailed *P* < .05 was considered statistically significant. All analyses were performed using Stata SE software, version 16.1 (StataCorp).

## Results

We identified a total of 16 765 incident cases of ICH (mean [SD] age, 72.8 [13.1] years; 8761 male [52.3%]) in Denmark during the study period. A total of 7473 (44.6%) were taking antithrombotic drugs at ICH onset. The 30-day case fatality of patients with ICH was 30.1% (eTable 2 in the Supplement).

Compared with 660 477 controls, cases had higher levels of comorbidity for all disorders included in the present analyses, including hypertension (OR, 1.47; 95% CI, 1.42-1.52), history of ischemic stroke (OR, 3.15; 95% CI, 3.02-3.28), atrial fibrillation (OR, 1.90; 95% CI, 1.81-1.99), and illnesses indicative of high alcohol use (OR, 2.35; 95% CI, 2.22-2.49) (eTable 2 in the Supplement). Preadmission medication use was more frequent among cases than controls for the drugs included as covariates (nonaspirin nonsteroidal anti-inflammatory drugs: 3221 [19.2%] cases vs 120 919 [18.3%] controls; selective serotonin reuptake inhibitors: 2317 [13.8%] cases vs 59 194 [9.0%] controls; statins: 4896 [29.2%] cases vs 177 460 [26.9%] controls; oral corticosteroids: 1201 [7.2%] cases vs 43 992 [6.7%] controls) except for hormone replacement therapy (1190 [14.9%] cases vs 50 989 [16.1%] controls) (eTable 2 in the Supplement).

Current use of low-dose aspirin (aOR, 1.51; 95% CI, 1.44-1.59), clopidogrel (aOR, 1.65; 95% CI, 1.47-1.84), DOAC (aOR, 1.83; 95% CI, 1.61-2.07), and VKA (aOR, 2.76; 95% CI, 2.58-2.96) was associated with higher risk of ICH. Associations of current use of individual antithrombotics with risk of ICH did not vary by sex; however, associations were stronger in younger age groups for all antithrombotics (eTable 3 in the Supplement). In analyses for individual anticoagulants, current use of all OACs except dabigatran (aOR, 0.82; 95% CI, 0.62-1.09) was associated with higher risk of ICH (Table 1). Associations of recency and duration of antithrombotic use with risk of ICH are presented in eTables 4 and 5 in the Supplement. Subanalyses by indication for OAC therapy returned similar results to main analyses for patients with atrial fibrillation; for patients with VTE, the risk of ICH was similar for DOACs and VKAs (eTable 6 in the Supplement).

**Table 1.** Current Use of Antithrombotics and Risk of Intracerebral Hemorrhage in Denmark, 2005-2018

Drug	No. (%) cases (n = 16 765)	No. (%) controls (n = 660 477)	OR (95% CI)^a^	Adjusted OR (95% CI)^b^
No antithrombotic drug^c^	8354 (49.8)	430 276 (65.1)	1 [Reference]	1 [Reference]
Current use of antithrombotic drug^d^				
Antiplatelet drug				
Low-dose aspirin^e^	4818 (28.7)	149 242 (22.6)	1.86 (1.78-1.93)	1.51 (1.44-1.59)
Clopidogrel^e^	1036 (6.2)	22 223 (3.4)	2.68 (2.50-2.88)	1.65 (1.47-1.84)
Anticoagulant drug^d^				
DOAC^e^^,^^f^	511 (3.0)	11 774 (1.8)	2.47 (2.23- 2.73)	1.83 (1.61-2.07)
Dabigatran	74 (0.4)	3922 (0.6)	1.07 (0.84-1.36)	0.82 (0.62-1.09)
Rivaroxaban	271 (1.6)	3973 (0.6)	3.85 (3.35-4.42)	2.96 (2.49-3.53)
Apixaban	165 (1.0)	3856 (0.6)	2.46 (2.07-2.93)	1.63 (1.30-2.05)
Edoxaban	5 (0.0)	96 (0.0)	3.04 (1.19-7.78)	4.55 (1.26-16.41)
VKA^e^	2009 (12.0)	33 275 (5.0)	3.49 (3.31-3.69)	2.76 (2.58-2.96)
Warfarin	1957 (11.7)	32 237 (4.9)	3.51 (3.32-3.71)	2.76 (2.57-2.96)
Current single antithrombotic drug use^g^^,^^h^				
Low-dose aspirin	3060 (18.3)	118 824 (18.0)	1.45 (1.39-1.52)	1.40 (1.33-1.48)
Clopidogrel	597 (3.6)	13 166 (2.0)	2.62 (2.39-2.87)	1.48 (1.31-1.67)
DOAC^i^	327 (2.0)	8039 (1.2)	2.30 (2.03-2.60)	1.80 (1.58-2.06)
VKA	1258 (7.5)	23 415 (3.5)	3.09 (2.89-3.30)	2.65 (2.46-2.85)
Current dual antithrombotic drug use^g^^,^^j^				
Low-dose aspirin and dipyridamole	667 (4.0)	11 427 (1.7)	3.29 (3.02-3.59)	1.49 (1.31-1.69)
Low-dose aspirin and clopidogrel	192 (1.1)	4667 (0.7)	2.26 (1.94-2.62)	1.56 (1.30-1.87)
Low-dose aspirin and DOAC^i^	35 (0.2)	665 (0.1)	2.91 (2.04-4.17)	2.51 (1.72-3.65)
Low-dose aspirin and VKA	486 (2.9)	6035 (0.9)	4.45 (4.02-4.93)	3.76 (3.33-4.25)
Clopidogrel and DOAC	7 (0.0)	119 (0.0)	3.52 (1.57-7.87)	1.69 (0.73-3.92)
Clopidogrel and VKA	20 (0.1)	200 (0.0)	6.03 (3.66-9.93)	3.69 (2.16-6.32)
Current triple antithrombotic use^g^^,^^k^				
Low-dose aspirin, clopidogrel and DOAC^i^	5 (0.0)	64 (0.0)	4.34 (1.65-11.43)	4.02 (1.45-11.14)
Low-dose aspirin, clopidogrel, and VKA	19 (0.1)	155 (0.0)	7.17 (4.27-12.04)	5.84 (3.34-10.22)

Abbreviations: DOAC, direct oral anticoagulant; OR, odds ratio; VKA, vitamin K antagonist.

^a^ Adjusted for age, sex, and calendar period (year) by design.

^b^ Adjusted for age, sex, and calendar period (by design) and the following, based on register data: hypertension, previous ischemic stroke, diabetes, chronic renal insufficiency, chronic hepatic disease, coagulopathy, heart failure, ischemic heart disease, peripheral artery disease, cancer, high alcohol consumption, chronic obstructive pulmonary disease, use of oral anticoagulants, low-dose aspirin, clopidogrel, other adenosine diphosphate inhibitors (ticagrelor or prasugrel), statins, nonsteroidal anti-inflammatory drugs, selective serotonin reuptake inhibitors, hormone replacement therapy, or oral corticosteroid drugs. In drug-specific analyses of anticoagulants use of oral anticoagulants individual variables for use of dabigatran, rivaroxaban, apixaban, edoxaban, or warfarin were adjusted for.

^c^ Nonuse of any antithrombotic drug is defined as no use of any antiplatelet or anticoagulant in the 12 months preceding the index date.

^d^ Within last 12 months before the index date.

^e^ Concurrent use or previous use (within 12 months before index date) of other antithrombotic drugs included.

^f^ Current users of more than 1 DOAC (<5 cases and 95 controls) (eg, due to switching, only contributed once in analyses of DOAC as a class of drugs).

^g^ Use 0 to 30 days before the index date.

^h^ Users of 2 or more antithrombotic drugs within 12 months of the index date are excluded.

^i^ Users of more than 1 type of DOAC in 12 months before the index date are excluded.

^j^ Users of 3 or more antithrombotic drugs within 12 months before the index date are excluded.

^k^ Users of 4 or more antithrombotic drugs within 12 months before the index date are excluded.

The risk of ICH associated with the use of antithrombotic drug combinations varied across regimens of treatment, with the lowest risk being associated with low-dose aspirin and dipyridamole (aOR, 1.49; 95% CI, 1.31-1.69) and the highest risk observed with triple therapy with VKAs, low-dose aspirin, and clopidogrel (OR, 5.84; 95% CI, 3.34-10.22) (Wald test for overall difference in effect estimates, *P* < .001) (Table 1).

The strongest association with fatal ICH was found with current use of VKAs (aOR, 4.41; 95% CI, 3.94-4.94) and the weakest with low-dose aspirin use (aOR, 1.99; 95% CI, 1.82-2.17). For nonfatal ICH, the strongest association was found with VKAs (aOR, 2.18; 95% CI, 2.00-2.38) and the weakest with clopidogrel (OR, 1.32; 95% CI, 1.15-1.51) (Table 2).

**Table 2.** Association of Current Antithrombotic Use With Fatal ICH (Died Within 30 Days of Onset) and Nonfatal ICH (Alive 30 Days After Onset)

Antithrombotic use	No. (%) of cases	No. (%) of controls	OR (95% CI)^a^	Adjusted OR (95% CI)^b^
Fatal ICH (n = 5038 cases and 199 238 controls)				
Nonuse of any antithrombotic drug^c^	1787 (35.5)	118 153 (59.3)	1 [Reference]	1 [Reference]
Current use^d^^,^^e^				
Low-dose aspirin	1852 (36.8)	53 265 (26.7)	2.58 (2.40-2.76)	1.99 (1.82-2.17)
Clopidogrel	411 (8.2)	7304 (3.7)	4.23 (3.75-4.78)	2.64 (2.18-3.20)
DOAC	182 (3.6)	3889 (2.0)	3.31 (2.75-3.98)	2.62 (2.09-3.29)
VKA	894 (17.7)	11 807 (5.9)	5.69 (5.20-6.23)	4.41 (3.94-4.94)
Nonfatal ICH (n = 11 727 cases and 461 239 controls)				
Nonuse of any antithrombotic drug^c^	6567 (56.0)	312 123 (67.7)	1 [Reference]	1 [Reference]
Current use^d^^,^^e^				
Low-dose aspirin	2966 (25.3)	95 977 (20.8)	1.60 (1.53-1.68)	1.33 (1.26-1.42)
Clopidogrel	625 (5.3)	14 919 (3.2)	2.17 (1.98-2.37)	1.32 (1.15-1.51)
DOAC	329 (2.8)	7885 (1.7)	2.18 (1.92-2.47)	1.59 (1.37-1.86)
VKA	1115 (9.5)	21 468 (4.7)	2.69 (2.50-2.88)	2.18 (2.00-2.38)

Abbreviations: DOAC, direct oral anticoagulant; ICH, intracerebral hemorrhage; OR, odds ratio; VKA, vitamin K antagonist.

^a^ Adjusted for age, sex, and calendar period (year) by design.

^b^ Adjusted for age, sex, and calendar period (by design) and the following, based on register data: hypertension, previous ischemic stroke, diabetes, chronic renal insufficiency, chronic hepatic disease, coagulopathy, heart failure, ischemic heart disease, peripheral artery disease, cancer, high alcohol consumption, chronic obstructive pulmonary disease, use of oral anticoagulants, low-dose aspirin, clopidogrel, other adenosine diphosphate inhibitors (ticagrelor or prasugrel), statins, nonsteroidal anti-inflammatory drugs, selective serotonin reuptake inhibitors, hormone replacement therapy, or oral corticosteroid drug.

^c^ Nonuse of any antithrombotic drug is defined as no use of any antiplatelet or anticoagulant in the 12 months preceding the index date.

^d^ Treatment episode ending 0 to 30 days before the index date.

^e^ Concurrent use or previous use (within 12 months before the index date) of other antithrombotic drugs included.

Analyses for individual antithrombotics restricted to the later study period (2014-2018) (eTable 7 in the Supplement) returned risk estimates that were similar to main analyses. Analyses of duration of naive use of individual DOACs had no major association with risk estimates (eTable 8 in the Supplement). In the late period analyses with current use of a VKA as reference group, risk of ICH was lower with current DOAC use (aOR, 0.78; 95% CI, 0.65-0.89) (eTable 9 in the Supplement). In corresponding analyses of individual DOACs with current use of warfarin as the reference group, dabigatran was associated with lowest risk of ICH (aOR, 0.34; 95% CI, 0.25-0.44), followed by apixaban (aOR, 0.73; 95% CI, 0.61-0.88), whereas rivaroxaban was associated with the highest risk of ICH (aOR, 1.20; 95% CI, 1.03-1.41). Standard dose of a DOAC was more strongly associated with ICH than reduced dose (aOR, 1.41; 95% CI, 1.16-1.70); however, in analyses of individual DOACs, no clear dose-response association was found (eTable 10 in the Supplement).

Data for this study did not include information on socioeconomic status, a potential confounder. However, in a similar Danish data set at our disposal (eMethods in the Supplement), we found that addition of education and income covariates to the main model had a minor association with the results (eTable 11 in the Supplement). Finally, in a negative control analysis^49^ (eMethods in the Supplement), current use of proton pump inhibitors was not associated with risk of ICH (aOR, 1.04; 95% CI, 0.99-1.09).

### Descriptive Analyses

We calculated the annual IR of ICH in Denmark in 2005 to 2018 (Figure; eTable 12 in the Supplement). Comparisons between the second and first half of the study period yielded IRRs of 0.87 (95% CI, 0.85-0.90) for ICH overall and 0.70 (95% CI, 0.66-0.74) for fatal ICH; corresponding estimates for those 85 years and older were 0.91 (95% CI, 0.85-0.98) for ICH overall and 0.70 (95% CI, 0.66-0.74) for fatal ICH (Table 3 ). In subanalyses, we calculated annual IRs using the Patient Registry as source for ICH cases (as opposed to the Stroke Registry in main analyses); we also calculated IRs for ICH in Western Denmark as explained in eFigures 1 and 2 in the Supplement. The IRRs based on these subanalyses produced results similar to main analyses. However, for patients 85 years and older, analyses of Western Denmark based on the Patient Registry produced IRRs above unity (eg, overall ICH: 1.13; 95% CI, 1.04-1.24) (eTable 13 in the Supplement). The regional subset analyses based on verified cases of spontaneous ICH in the Region of Southern Denmark returned estimates of IRRs indicative of no change in IRs in the period examined (2009-2017); for those 85 years and older, point estimates for IRRs in the latest period (2015-2017) were above unity (1.25; 95% CI, 1.00-1.56) (eTable 14 and eFigure 3 in the Supplement).

**Figure.**
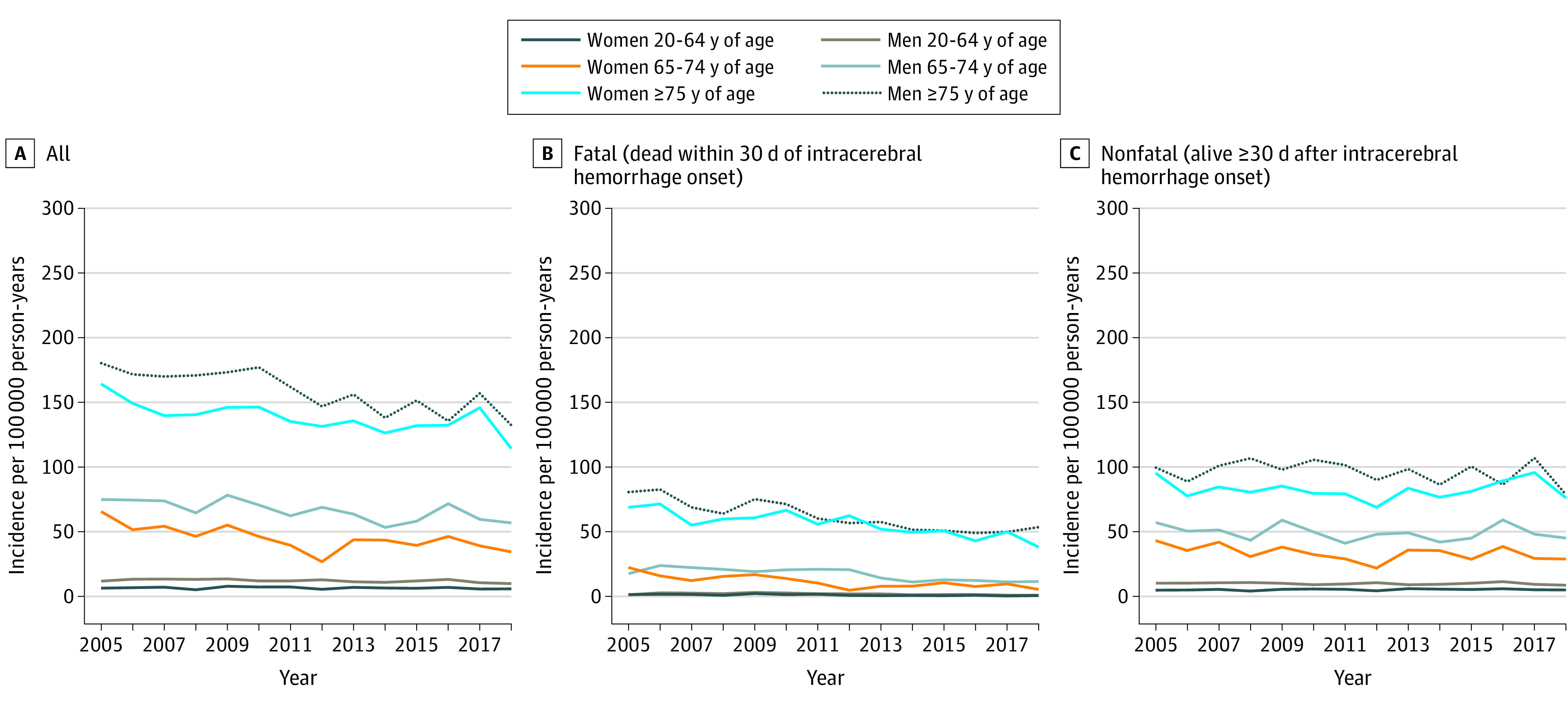
Incidence Rate of Intracerebral Hemorrhage in Denmark by Sex and Age Group, 2005-2018

**Table 3.** Incidence Rate of ICH per 100 000 Person-years

Age group, y	2005-2011		2012-2018		Incidence rate ratio (95% CI)^b^	*P* value
	No. of cases	sIR (95% CI)^a^	No. of cases	sIR (95% CI)^a^		
All ICH						
All	8450	29.9 (29.3-30.5)	8315	26.2 (25.6-26.7)	0.87 (0.85-0.90)	<.001
20-64	2236	9.8 (9.4-10.2)	2044	9.0 (8.6-9.4)	0.92 (0.86-0.97)	.005
65-74	2024	60.2 (57.8-62.8)	2166	49.2 (47.1-51.5)	0.82 (0.77-0.87)	<.001
75-84	2756	142 (137-147)	2659	123 (118-128)	0.87 (0.82-0.91)	<.001
≥85	1434	193 (183-203)	1446	176 (167-185)	0.91 (0.85-0.98)	.01
Fatal ICH^c^						
All	2818	10.0 (9.6-10.3)	2220	7.0 (6.7-7.3)	0.70 (0.66-0.74)	<.001
20-64	446	2.0 (1.8-2.1)	252	1.1 (1.0-1.3)	0.57 (0.49-0.66)	<.001
65-74	596	17.6 (16.4-19.0)	451	10.3 (9.3-11.3)	0.58 (0.51-0.66)	<.001
75-84	1112	57.2 (54.0-60.7)	1517	40.4 (37.7-43.3)	0.71 (0.64-0.77)	<.001
≥85	664	89.7 (83.2-96.6)	650	78.7 (72.7-85.2)	0.88 (0.79-0.98)	.02

Abbreviations: ICH, intracerebral hemorrhage; sIR, standardized incidence rate.

^a^ Age (5-year bands) and sex standardized to 2011 population in Denmark.

^b^ Second half (2012-2018) vs first half (2005-2011) of study period.

Compared with 2005, current drug use among controls in 2018 was higher for antithrombotics overall (27.6% vs 31.7%), for OACs (overall: 3.8% vs 11.1%; DOACs: 0% vs 7.0%; VKAs: 3.8% vs 4.2%), and for clopidogrel (1.0% vs 6.8%) but was lower for antiplatelet drugs overall (24.7% vs 21.4%) because of lower use of low-dose aspirin (24.3% vs 15.3%) (eTable 15 in the Supplement). We observed similar patterns in analyses stratified by age and sex (eg, among men ≥85 years of age for 2005 vs 2018: antithrombotics overall: 42.8% vs 56.9%; OACs: 4.5% vs 24.3%; DOACs: 0% vs 14.5%; VKAs: 4.5% vs 10%; antiplatelet drugs: 39.2% vs 34.4%; low-dose aspirin: 38.6% vs 23.9%; clopidogrel: 1.0% vs 11.5%). Corresponding age- and sex-standardized figures within the first and second parts of the study period are presented in Table 4.

**Table 4.** Prevalence of Use of Antithrombotic Drugs in the General Population Controls in 2005-2011 vs 2012-2018^a^

Age group, y	2005-2011		2012-2018		Prevalence ratio (95% CI)^c^	*P* value
	No. of controls	Standardized prevalence, %^b^	No. of controls	Standardized prevalence, %^b^		
Any oral anticoagulant^d^						
All	16 022	4.8	28 779	8.6	1.78 (1.74-1.81)	<.001
20-64	1130	1.3	1211	1.6	1.19 (1.10-1.29)	<.001
65-74	3532	4.4	5630	6.4	1.46 (1.40-1.52)	<.001
75-84	8062	7.4	13 484	12.6	1.71 (1.66-1.75)	<.001
≥85	3298	5.7	8454	14.5	2.52 (2.43-2.62)	<.001
Vitamin K antagonist						
All	15 969	4.8	17 306	5.2	1.08 (1.06-1.11)	<.001
20-64	1128	1.3	803	1.0	0.79 (0.72-0.87)	<.001
65-74	3513	4.4	3433	4.0	0.91 (0.87-0.95)	<.001
75-84	8036	7.4	8392	8.0	1.08 (1.05-1.11)	<.001
≥85	3292	5.7	4678	8.1	1.41 (1.35-1.47)	<.001
Direct oral anticoagulant						
All	60	0	11 714	3.4	196 (152-251)	<.001
20-64^e^	<5	0	415	0.5	234 (58-947)	<.001
65-74^e^	<25	0	2241	2.5	99 (66-151)	<.001
75-84	29	0	5207	4.8	180 (125-258)	<.001
≥85	7	0	3851	6.6	560 (267-1176)	<.001
Any antiplatelet drug^f^						
All	87 638	26.4	78 325	23.7	0.90 (0.89-0.90)	<.001
20-64	7501	8.7	5954	7.7	0.88 (0.86-0.91)	<.001
65-74	19 236	24	18 414	21.5	0.90 (0.88-0.91)	<.001
75-84	37 908	34.7	33 083	31.5	0.91 (0.90-0.92)	<.001
≥85	22 993	40.2	20 874	36.2	0.90 (0.89-0.91)	<.001
Low-dose aspirin						
All	85 133	25.6	64 109	19.4	0.76 (0.75-0.77)	<.001
20-64	7263	8.4	5006	6.4	0.77 (0.74-0.79)	<.001
65-74	18 689	23.3	15 344	18	0.77 (0.76-0.79)	<.001
75-84	36 749	33.6	27 004	25.8	0.77 (0.76-0.78)	<.001
≥85	22 432	39.3	16 755	29.2	0.74 (0.73-0.75)	<.001
Clopidogrel						
All						
20-64	578	0.7	1212	1.6	2.34 (2.12-2.58)	<.001
65-74	1210	1.5	3760	4.3	2.87 (2.70-3.06)	<.001
75-84	2197	2.0	7317	6.8	3.39 (3.24-3.56)	<.001
≥85	1091	1.9	4858	8.3	4.41 (4.14-4.70)	<.001

Abbreviation: ICH, intracerebral hemorrhage.

^a^ Controls were randomly selected among individuals in the general population who matched ICH cases with regard to age, sex, and calendar time (index date, see text). Current use defined as treatment episode with the drug within a month (0-30 days) of the date of selection as control.

^b^ Age (5-year bands) and sex standardized to 2011 population in Denmark.

^c^ Second half (2012-2018) vs first half (2005-2011) of study period.

^d^ Vitamin K antagonist or direct oral anticoagulant.

^e^ Exact numbers not reported for strata with less than 5 patients (and here also for another connected sparse stratum) in accordance with Danish National Board of Data regulations.

^f^ Low-dose aspirin or clopidogrel.

We also calculated annual rates of current use of OACs among ICH cases (eFigure 4 in the Supplement). In 2018, the last year of the study period, 20.2% of cases were current users of OACs (DOACs: 12.9%; VKAs: 7.5%) and 26.4% were current users of antiplatelet drugs. Among men with ICH 85 years or older, the corresponding percentages were 31.1% for OACs (DOACs: 18.9%; VKAs: 12.2%) and 33.8% for antiplatelet drugs.

## Discussion

In this case-control study that included 16 765 patients with ICH, antithrombotic treatment was associated with higher risk of ICH. The risk was highest for a VKA, particularly when combined with single or dual antiplatelet drug therapy. The lowest risk of ICH was seen with single antiplatelet therapy. Direct oral anticoagulants were associated with an intermediate risk between antiplatelet drugs and VKAs. The OR of fatal ICH associated with antithrombotic drug use was lowest for low-dose aspirin, intermediate for clopidogrel and DOACs, and highest for VKAs.

Compared with warfarin, dabigatran and apixaban were associated with a lower risk of ICH, whereas rivaroxaban was associated with a risk of ICH similar to warfarin’s. These results are in line with the only previous study^2^ of ICH risk in association with OAC use in the wider population. In addition, these findings are similar to those reported in some studies^15,23,25,31^ of the risk of intracranial hemorrhage with OAC use, including 2 recent large observational studies^24,50^ of DOAC safety and effectiveness; however, most of these studies^24,25,31,50^ found that all DOACs, including rivaroxaban, had lower risk estimates than warfarin.

This study found that a higher (ie, standard) dose of a DOAC was associated with a higher risk of ICH than reduced DOAC dose. To our knowledge, no other observational studies have assessed the risk of ICH by dose. The results of this study on the association between DOAC dose and risk of ICH should be interpreted with caution because unmeasured factors (ie, other than standard recommendations) probably influence choice of dose.^51^ We note, however, that our findings are in line with the results on risk of intracranial hemorrhage reported in 2 randomized clinical trials^52,53^ that specifically addressed dose-response effects.

This article presents novel data on temporal changes in the incidence of ICH and associated changes in patterns of antithrombotic drug use, including DOACs, whereas previous studies^38,39,54^ of this issue focused exclusively on the role of warfarin. The IR of ICH (overall and fatal) decreased within the 14-year study period despite the increased use of OACs, including among individuals 75 years and older, who were the age group most frequently treated with OACs. Improved awareness and control of hypertension in recent decades probably plays a central role in the observed decrease in ICH incidence rates.^38,55^ However, a major part of this decrease occurred in the first half of the study period, whereas temporal trends with respect to increased use of OACs were more marked in the second half of the study period. Our comparisons of these 2 periods indicate that the IR of ICH may have leveled in recent years or even increased slightly among older patients, coinciding with more widespread use of anticoagulants. Whatever the case, it is imperative that these findings be held up against the known substantial net clinical benefits of antithrombotic drugs in patients with clear therapeutic indications.^3,56,57,58^

### Strengths and Limitations

This study has strengths. Its main strength is its large size and its setting, where access to the health care system is independent of income level. The administrative registers provided complete coverage of prospectively collected data on all Danish residents, whereby recall bias was eliminated and selection bias minimized.

The study also has limitations. The Stroke Registry was used for case ascertainment because approximately 80% of cases recorded in this source were spontaneous ICHs,^48^ the focus of the study. The estimates of IRs for spontaneous ICH based on the Stroke Registry data were in line with previous reports^59,60,61^ from high-income countries with predominantly White populations. However, according to a recent validation study, 21% of spontaneous ICH cases were not recorded in the Stroke Registry, and the sensitivity of this source was reported to have decreased over time.^48^ Supplementary analyses were therefore conducted based on the Patient Registry, which is reported to have a more stable sensitivity but a lower positive predictive value than the Stroke Registry.^48^ The findings of these supplemental analyses were compatible with a decrease in IRs of ICH in the study period. Nevertheless, the decrease observed in the main analyses could possibly be due to the above-mentioned temporal changes of sensitivity of the Stroke Registry. Of note, analyses of the regional subset, based on verified cases of spontaneous ICH, indicated largely unchanged IRs for 2009 to 2017 (overall and for those 75 years and older) and a possible slight increase in IRs among those 85 years and older.

Small numbers prevented us from assessing the association of use of prasugrel and ticagrelor with the risk of ICH and from evaluating edoxaban as extensively as other DOACs. Data on international normalized ratio were lacking^29^; therefore, this factor’s influence on the risk of ICH among patients using a VKA could not be assessed. Data on tobacco and alcohol consumption were also lacking. However, high alcohol use was classified based on register data, an approach demonstrated to produce results similar to those when medical records are used as the source of information.^62^ Because this study is observational, residual confounding by factors included in our model and unmeasured potential confounders cannot be ruled out.

## Conclusions

In Denmark, use of DOACs was associated with lower risk of ICH than use of a VKA. A marked increase in OAC use in the study period can be nearly all ascribed to increased DOAC use and does not appear to be associated with an increase in incidence of ICH. However, practitioners now face a higher proportion of older patients with OAC-associated ICH. Because ICH is associated with significant mortality, strategies for reducing occurrence and impact are needed.

## Notes

Supplement. **eMethods.** Supplemental Methods **eReferences** **eTable 1.** List of Codes Used to Retrieve and Classify Data for the Study **eTable 2.** Characteristics of Cases With Incident Intracerebral Hemorrhage and Their General Population Controls, Denmark 2005-2018 **eTable 3.** Association of Antithrombotic Drug Use With Intracerebral Hemorrhage Stratified by Age and Sex **eTable 4.** Use of Antiplatelet Drugs and Risk of Intracerebral Hemorrhage In Denmark Stratified by Recency and Duration of Current Use, 2005-2018 **eTable 5.** Use of Anticoagulant Drugs and Risk of Intracerebral Hemorrhage in Denmark Stratified by Recency and Duration of Current Use, 2005-2018 **eTable 6.** Current Use of Anticoagulants in Patients With Atrial Fibrillation and Venous Thromboembolism and Risk of Intracerebral Hemorrhage in Denmark, 2005-2018 **eTable 7.** Current Use of Antithrombotic Drugs and Risk of Intracerebral Hemorrhage in Denmark, 2014-2018 **eTable 8.** Duration of Current Naive Use of Specific Anticoagulants and Risk of Intracerebral Hemorrhage in Denmark, 2014-2018 **eTable 9.** Current Use of DOAC vs Current Use of VKA and Risk of Intracerebral Hemorrhage in Denmark **eTable 10.** Dose of Current Use of Specific Anticoagulants and Risk of Intracerebral Hemorrhage in Denmark, 2014-2018 **eTable 11.** Odds Ratios for Association of Antithrombotic Drug Use in Models With and Without Adjustment for Socioeconomic Status, Denmark 2005-2016 **eTable 12.** Annual Number of Cases and Incidence Rate of Intracerebral Hemorrhage per 100,000 Person-years in Denmark, 2005-2018 **eTable 13.** Incidence Rate Ratio of Intracerebral Hemorrhage in 2005–2011 Versus 2012–2018 Using Stroke Registry and Patient Registry – for Entire Danish Population (5.8 Million) and Limited to Population of Western Denmark (3.2 Million) **eTable 14.** Incidence Rate of Verified Spontaneous Intracerebral Hemorrhage per 100,000 Person-years and Prevalence of Use of Antithrombotic Drugs Among General Population Controls in Region of Southern Denmark, 2009-2017 **eTable 15.** Percentage of Cases and Their General Population Controls Classified as Current Users of Antithrombotic Drugs, Denmark 2005-2018 **eFigure 1.** Incidence Rate of Intracerebral Hemorrhage in Denmark by Source Used to Identify Cases, Denmark, 2005-2018 **eFigure 2.** Incidence Rate of Intracerebral Hemorrhage by Source Used to Identify Cases, Western Denmark, 2005-2018 **eFigure 3.** Standardized Incidence Rates of Verified Spontaneous Intracerebral Hemorrhage (ICH) and Prevalence of Antithrombotic Drug Use in Region of Southern Denmark, 2009-2017 **eFigure 4.** Annual Percentage of Current Use of Antithrombotic Drugs Among Cases With Intracerebral Hemorrhage and Their General Population Controls, Denmark 2005-2018
