# Error in Author Name and Affilations

**DOI:** 10.1001/jamanetworkopen.2021.18300

**Published:** 2021-06-17

In the Original Investigation titled “Trends in Incidence of Intracerebral Hemorrhage and Association With Antithrombotic Drug Use in Denmark, 2005-2018,”^1^ published May 5, 2021, Dr Al-Shahi Salman’s surname was given incorrectly in the Article Information. This article has been corrected.^1^
