# Supplementary material for: Trends in Incidence of Intracerebral Hemorrhage and Association With Antithrombotic Drug Use in Denmark, 2005-2018
Source: JAMA Netw Open. 2021 May 5;4(5):e218380. doi: 10.1001/jamanetworkopen.2021.8380 (PMC8100864; doi:10.1001/jamanetworkopen.2021.8380)
Supplement: Supplement. — eMethods. Supplemental Methods eReferences eTable 1. List of Codes Used to Retrieve and Classify Data for the Study eTable 2. Characteristics of Cases With Incident Intracerebral Hemorrhage and Their General Population Controls, Denmark 2005-2018 eTable 3. Association of Antithrombotic Drug Use With Intracerebral Hemorrhage Stratified by Age and Sex eTable 4. Use of Antiplatelet Drugs and Risk of Intracerebral Hemorrhage In Denmark Stratified by Recency and Duration of Current Use, 2005-2018 eTable 5. Use of Anticoagulant Drugs and Risk of Intracerebral Hemorrhage in Denmark Stratified by Recency and Duration of Current Use, 2005-2018 eTable 6. Current Use of Anticoagulants in Patients With Atrial Fibrillation and Venous Thromboembolism and Risk of Intracerebral Hemorrhage in Denmark, 2005-2018 eTable 7. Current Use of Antithrombotic Drugs and Risk of Intracerebral Hemorrhage in Denmark, 2014-2018 eTable 8. Duration of Current Naive Use of Specific Anticoagulants and Risk of Intracerebral Hemorrhage in Denmark, 2014-2018 eTable 9. Current Use of DOAC vs Current Use of VKA and Risk of Intracerebral Hemorrhage in Denmark eTable 10. Dose of Current Use of Specific Anticoagulants and Risk of Intracerebral Hemorrhage in Denmark, 2014-2018 eTable 11. Odds Ratios for Association of Antithrombotic Drug Use in Models With and Without Adjustment for Socioeconomic Status, Denmark 2005-2016 eTable 12. Annual Number of Cases and Incidence Rate of Intracerebral Hemorrhage per 100,000 Person-years in Denmark, 2005-2018 eTable 13. Incidence Rate Ratio of Intracerebral Hemorrhage in 2005–2011 Versus 2012–2018 Using Stroke Registry and Patient Registry – for Entire Danish Population (5.8 Million) and Limited to Population of Western Denmark (3.2 Million) eTable 14. Incidence Rate of Verified Spontaneous Intracerebral Hemorrhage per 100,000 Person-years and Prevalence of Use of Antithrombotic Drugs Among General Population Controls in Region of Southern Denmark, 2009-2017 eTable 15. P [file jamanetwopen-e218380-s001.pdf]

Supplementary Online Content

Hald SM, Möller S, García Rodríguez LA, et al. Trends in incidence of intracerebral hemorrhage and association with antithrombotic drug use in Denmark, 2005-2018. *JAMA Netw Open*. 2021;4(5):e218380. doi:10.1001/jamanetworkopen.2021.8380

eMethods. Supplemental Methods

eReferences

eTable 1. List of Codes Used to Retrieve and Classify Data for the Study

eTable 2. Characteristics of Cases With Incident Intracerebral Hemorrhage and Their General Population Controls, Denmark 2005-2018

eTable 3. Association of Antithrombotic Drug Use With Intracerebral Hemorrhage Stratified by Age and Sex

eTable 4. Use of Antiplatelet Drugs and Risk of Intracerebral Hemorrhage In Denmark Stratified by Recency and Duration of Current Use, 2005-2018

eTable 5. Use of Anticoagulant Drugs and Risk of Intracerebral Hemorrhage in Denmark Stratified by Recency and Duration of Current Use, 2005-2018

eTable 6. Current Use of Antithrombotics in Patients With Atrial Fibrillation and Venous Thromboembolism and Risk of Intracerebral Hemorrhage in Denmark, 2005-2018

eTable 7. Current Use of Antithrombotic Drugs and Risk of Intracerebral Hemorrhage in Denmark, 2014-2018

eTable 8. Duration of Current Naive Use of Specific Anticoagulants and Risk of Intracerebral Hemorrhage in Denmark, 2014-2018

eTable 9. Current Use of DOAC vs Current Use of VKA and Risk of Intracerebral Hemorrhage in Denmark

eTable 10. Dose of Current Use of Specific Anticoagulants and Risk of Intracerebral Hemorrhage in Denmark, 2014-2018

eTable 11. Odds Ratios for Association of Antithrombotic Drug Use in Models With and Without Adjustment for Socioeconomic Status, Denmark 2005-2016

eTable 12. Annual Number of Cases and Incidence Rate of Intracerebral Hemorrhage per 100,000 Person-years in Denmark, 2005-2018

eTable 13. Incidence Rate Ratio of Intracerebral Hemorrhage in 2005–2011 Versus 2012–2018 Using Stroke Registry and Patient Registry – for Entire Danish Population (5.8 Million) and Limited to Population of Western Denmark (3.2 Million)

eTable 14. Incidence Rate of Verified Spontaneous Intracerebral Hemorrhage Per 100,000 Person-years and Prevalence of Use of Antithrombotic Drugs Among General Population Controls in Region of Southern Denmark (1.2 Million), 2009-2017

eTable 15. Percentage of Cases and Their General Population Controls Classified as Current Users of Antithrombotic Drugs, Denmark 2005-2018

eFigure 1. Incidence Rate of Intracerebral Hemorrhage in Denmark by Source Used to Identify Cases, Denmark, 2005-2018

eFigure 2. Incidence Rate of Intracerebral Hemorrhage by Source Used to Identify Cases
Northern, Mid, and Southern Regions of Denmark, 2005-2018

eFigure 3. Standardized Incidence Rates of Verified Spontaneous Intracerebral Hemorrhage (ICH) and Prevalence of Antithrombotic Drug Use in Region of Southern Denmark, 2009-2017

eFigure 4. Annual Percentage of Current Use of Antithrombotic Drugs Among Cases With Intracerebral Hemorrhage and Their General Population Controls, Denmark 2005-2018

This supplementary material has been provided by the authors to give readers additional information about their work.

© 2021 Hald SM et al. *JAMA Network Open.*

eMethods. Supplement Methods

Assessment of antithrombotic drug exposure

Information on prescriptions dispensed at community pharmacies in Denmark have been prospectively recorded in the Danish National Prescription Registry (Prescription Registry) since 1995. For each prescription, the date of dispensing and a full account of the dispensed product, including the anatomical therapeutic chemical (ATC) code, are recorded. The indication and prescribed dose are not available in the Prescription Registry.

To calculate date of end of supply of each prescription, we set each prescription to last the number of days that corresponded to the number of pills dispensed (divided by 2 for dipyridamole, dabigatran, apixaban, and edoxaban and 1.5 for warfarin[1]). Between pairs of consecutive prescriptions, we allowed a grace period of 60 days between the end of supply of the first prescription of that pair and the date of the next prescription. An episode of treatment comprised consecutive pairs of prescriptions presented without a gap. Based on the most recent treatment episode before the index date, we divided exposure by recency of use as described in the main text. However, as we observed increased ORs associated with past use of certain antithrombotics, we also performed analyses where past use (91-365 days before index date in main analysis) was subclassified into two categories (91-183 days vs 184-365 days before index date).

For analyses of duration of use, we subdivided current use according to the duration of the treatment episode into <1 month; ≥1 month to ≤3 months; >3 months to ≤12 months; >1 year to ≤3 years; and >3 years.

We calculated the risk of intracerebral hemorrhage (ICH) among current users with concurrent multiple use of antithrombotics (dual therapy with antiplatelets, or antiplatelets combined with anticoagulant). In these analyses, as explained above, use of dipyridamole was disregarded (except in the analysis concerning dual therapy with low-dose aspirin (ASA) and dipyridamole). In the analysis of current dual therapy, individuals who had used any other antithrombotic than the two in question within the 12-month period preceding the index date were not retained as dual current users. The same strategy was adopted for current triple therapy (i.e., only users of the three drugs in question within the 12-month period were included in that particular analysis).

Some subjects switch between antithrombotic drugs. To analyse the effect of current use of antithrombotic drugs in monotherapy, we therefore repeated analyses comparing current users who had only used a single antithrombotic drug (or class of drugs, depending on analysis) within 12 months before index date with non-users. Therefore, we excluded users with use of more than one antithrombotic drug in the last 12 months from these analyses. We did not analyze the effect of single dipyridamole use (with no concurrent use of ASA), since, in accordance with Danish guidelines on stroke prevention, this drug is only recommended in combination with ASA.[2,3] Accordingly, we only studied the combination ASA/dipyridamole.

We also performed analyses performed restricted to "new users" (also known as new initiators) of antithrombotics and "naive users" of oral anticoagulants. We defined "new users" as patients with only a single episode of use of the drug class (e.g. vitamin K antagonist (VKA)), or specific drug (e.g. warfarin) in question, who, furthermore, had no recorded use of the drug class/specific drug in the years 1995 to 1996; the latter criterion was used to minimize misclassification of prevalent use within the first 2 years after the Prescription Registry became operational in 1995. We defined "naive use" of oral anticoagulants (i.e. no recorded previous use of other oral anticoagulants) at group level (e.g., direct oral anticoagulant (DOAC) use by patient with no previous use of VKA) and at drug-specific level (e.g., dabigatran use by patient with no previous use of any other DOAC or VKA).

© 2021 Hald SM et al. *JAMA Network Open.*

For DOACs, we calculated the effect of dose of the drug on risk of ICH. Based on capsule or tablet strength of the most recent prescription presented before the index date, we classified daily dose into standard (dabigatran 300 mg, apixaban 10 mg, rivaroxaban 20 mg, edoxaban 60 mg), or reduced (dabigatran ≤220 mg, apixaban ≤5 mg, rivaroxaban ≤15 mg, edoxaban≤30mg).

With few exceptions (see below), we used a common reference group of exposure. This reference group comprised subjects with non-use of antithrombotic drugs (end of supply of most recent prescription of any antithrombotic drug before index date stopped more than 365 days before index date, or no recorded use of any antithrombotic drug since 1995).

We wished to capture recent developments in use of oral anticoagulants and therefore, we performed analyses with data limited to 2014-2018. In these analyses we also calculated risk estimates using current use of VKA (warfarin in drug-specific analyses) to better mirror the choices of comparator in previous clinical trials and observational research.

Potential confounders

We classified disorders that we regarded as potential confounders based on diagnosis codes from in-patient (1977 to 1 day prior to index date) and out-patient (1995 to 1 day prior to index date) contacts at hospitals in Denmark, prescriptions (1995 to 1 day prior to index date), or a combination of diagnosis codes and prescriptions, see eTable 1. We also classified exposure to certain drugs (in separate variables: NSAIDs, SSRIs, statin, hormone replacement therapy, oral corticosteroids) we regarded as potential confounders based on prescription data for the period 1 year to 1 day prior to index date.

Fatal ICH

Date of death of patients with ICH was established through linkage with the Civil Registration System[4] which is continuously updated with regard to migration and vital status of all citizens in Denmark. We classified patients who died within 30 days of ICH onset as cases of fatal ICH.

Statistical analyses

Analyses of associations of antithrombotic drug use with risk of ICH

For main analyses of use of antithrombotic drugs with risk of ICH, see manuscript.

Supplementary analyses of influence of socioeconomic status as potential confounder

We performed several supplementary analyses that are mainly presented in the manuscript. Here, we focus on the evaluation of influence of socioeconomic status as potential confounder

The main analyses are based on data up to 2018 held at the Danish Health Data Authority (DHDA) (in Danish, *Sundhedsdatastyrelsen*). However, the registries at DHDA do not hold information on socioeconomic status (SES), a potentially confounding factor. Therefore, we performed separate analyses of a dataset we built with data held by another vendor (Statistics Denmark) that included information on SES at the level of the individual but was only updated to 2016 (i.e. lacked data for 2017 and 2018). Importantly, all other data used in the analysis of this additional dataset were retrieved from copies of the same registries as held at DHDA and using the same codes as in the main analyses. We used this additional dataset to perform key analyses where we compared ORs for ICH associated with antithrombotic drug use with

© 2021 Hald SM et al. *JAMA Network Open.*

and without addition of two separate covariates concerning education level and income (ascertained the year before ICH onset) to the full model (for description of full model, see main text). This allowed us to gauge the direction and magnitude of confounding caused by SES.

Supplementary analysis of negative exposure controls

Use of negative exposure controls has been advocated as a tool for detecting confounding and bias in observational research.[5] For this purpose, we chose current use of proton pump inhibitors (PPI), as we had no a priori biologically founded hypothesis of a causal association between use of PPI and risk of ICH. In Denmark, in the study period, 97%-99% of the total quantity of PPI sold was dispensed on prescription (www.medstat.dk/en). We calculated recency and duration of PPI use as described above using dispensed defined daily doses (DDD)[6] to calculate the supply of individual prescriptions of PPI. We calculated the OR of current use of PPI after adjusting for all variables included in the main model.

Descriptive analyses

Annual incidence rates (IRs) of ICH and fatal ICH (overall and by sex) per 100,000 person-years were calculated using the number of ICH events as numerator and person-years at risk as denominator. Confidence intervals (CIs) were derived assuming a Poisson distribution. Standardized IRs (sIRs) were age- (5-year bands) and sex-standardized to the Danish population in 2011 using census data from Statistics Denmark. Using Poisson regression, the trend was calculated in the incidence rate of ICH of nationwide data (2005 to 2018). Trends in the 30-day case fatality rate of ICH patients were also tested.

Supplementary descriptive analyses

We repeated all descriptive analyses using the Patient Registry as the source, as no temporal trends in the high sensitivity of this registry for ICH diagnosis were observed in a recent validation study.[7] Further, as the introduction of a new electronic health record system in 2016 in hospitals in Eastern Denmark[8] could influence the completeness of the data, we also conducted analyses after excluding data from this region (i.e. with data from Western Denmark only).

<u>Analyses of regional subset with verified diagnoses</u>

We had access to data on all first-ever cases of spontaneous ICH in the Region of Southern Denmark (RSD; population 1.2 million) in 2009 through 2017.[5] In brief, we traced all adults (aged 20-99 years) in RSD with a first-ever diagnosis of ICH according to multiple sources (i.e., recorded admission in the Stroke Registry or any type of hospital contact recorded in the Patient Registry (admission, outpatient or emergency room)) in 2009-2017. For all thus identified potential cases, we retrieved medical record information (primarily discharge summaries and brain imaging study reports), based on which study physicians verified diagnoses of spontaneous ICH. Using this regional verified subset, we calculated sIRs (standardized to year 2011 as described above) of spontaneous ICH for 3-year time-bands (i.e., 2009-2011; 2012-2014; 2015-2017) and incidence rate ratios (IRRs; 2012-2014 vs 2009-2011; 2015-2017 vs 2009-2011). We could verify spontaneous ICH diagnosis in 2,556 patients. The positive predictive value (PPV) of a diagnosis of ICH varied by source and type of contact.[5] A small number of untraced records (n=188) were primarily due to paper medical records not being archived at some hospitals in the region. The distribution of untraced records was heavily skewed over time (2009-2011: 115; 2012-2014: 54; 2013-2015: 19). Therefore, we calculated sIRs by applying multiple imputation, i.e., 100 imputed

© 2021 Hald SM et al. *JAMA Network Open.*

data sets for the 188 patients with untraced status in the regional subset. We based imputation on published PPVs by calendar period.[5] We likewise calculated sIRs of fatal ICH based on verified cases after establishing 30-day mortality based on Civil Registration System data, as described above (see Fatal ICH).

We also computed the prevalence of current use of antithrombotic drugs among general controls that resided in RSD in 2009-2017 and could be matched to verified ICH cases on index date, age and sex. Based on these controls, we calculated percentage of users (age- and sex-standardized to year 2011) of antiplatelet and anticoagulant drugs within each 3-year time-band and prevalence ratios (i.e., 2012-2014 vs 2009-2011 and 2015-2017 vs 2009-2011).

© 2021 Hald SM et al. *JAMA Network Open.*

eReferences

1. Henriksen DP, Stage TB, Hansen MR, Rasmussen L, Damkier P, Pottegård A. The potential drug-drug interaction between proton pump inhibitors and warfarin. *Pharmacoepidemiol Drug Saf*. 2015;24(12):1337-1340. doi:10.1002/pds.3881

2. Ref.program. Referenceprogram for behandling af patienter med apopleksi og TCI (2013). Published online 2013. http://www.dsfa.dk/wpcontent/ uploads/REFERENCEPROGRAMFINAL20131.pdf

3. The Danish Council for the Use of Expensive Hospital Medicines (RADS). Baggrundsnotat for tromboseprofylakse som sekundær profylakse ved iskæmisk apopleksi. Published online 2016. https://www.regioner.dk/media/2077/bgn-inkl-bilag-apopleksi-feb-2016.pdf

4. Pedersen CB. The Danish Civil Registration System. *Scand J Public Health*. 2011;39(7 Suppl):22-25. doi:10.1177/1403494810387965

5. Lipsitch M, Tchetgen Tchetgen E, Cohen T. Negative controls: a tool for detecting confounding and bias in observational studies. *Epidemiology*. 2010;21(3):383-388. doi:10.1097/EDE.0b013e3181d61eeb

6. WHO Collaborating Centre for Drug Statistics Methodology. *Guidelines for ATC Classification and DDD Assignment.* WHO; 2020. http://www.whocc.no/atc_ddd_index/. 2020

7. Hald S, Sloth C, Agger M, et al. The validity of intracerebral hemorrhage diagnoses in the Danish Patient Registry and the Danish Stroke Registry. *Clinical Epidemiology*. 2020;12:1313-1326. doi:https://doi.org/10.2147/CLEP.S267583

8. Landex N. [The Epic healthcare system in Denmark]. *Ugeskr Laeg*. 2017;179(50).

© 2021 Hald SM et al. *JAMA Network Open.*

eTable 1. List of codes used to retrieve and classify data for the study

	**Hospital contact code or procedure code**	**Drug (ATC-code)**
**Outcome of interest**		
Intracerebral hemorrhage (ICH)	ICD-10: I61<br>[ICD-8: 431.00, 431.08, 431.09, 431.90, 431.98, 431.99; only used to exclude prevalent cases]	--
**Exposures of interest**		
Antiplatelet drugs[1]		
Aspirin – low dose	--	B01AC06 – acetylsalicylic acid (75 mg, 100 mg, or 150 mg per tablet)<br>B01AC30 – acetylsalicylic acid (50 mg per tablet) in combination with dipyridamole
Dipyridamole	--	B01AC07 (100 mg per tablet, or 200 mg per tablet)
Clopidogrel	--	B01AC04 (75 mg per tablet)
Other ADP drugs	--	B01AC22 – prasugrel (5 mg, or 10 mg per tablet)<br>B01AC24 - ticagrelor (60 mg, or 90 mg per tablet)
Anticoagulant drugs[1]		
Vitamin K antagonists	--	B01AA
Direct oral anticoagulants (DOAC)		
Dabigatran etexilate	--	B01AE07
Rivaroxaban	--	B01AF01
Apixaban	--	B01AF02
Edoxaban	--	B01AF03
**Covariates – Disorders**		
Hypertension[1,2]	ICD-8: 400-404<br>ICD-10: I10-I15	C03A, C08CA, C08DB01, C09A-D
Ischemic stroke[2]	ICD-8 : 433, 434, 436.01, 436.09, 436.90<br>ICD-10: I63, I64, I693	--
Diabetes[1,2]	ICD-8: 249, 250<br>ICD-10: E10-E14	A10
Chronic renal insufficiency[2]	ICD-8: 593.2	--
	ICD-10: N18 (excluding N181), N19, Z992, Z940	--
Chronic hepatic diseases[2]	ICD-8: 571.11, 571.19, 571.90, 571.91, 571.92, 571.93, 571.94, 571.99, 573<br>ICD-10: K71-K77	--
Coagulopathy[2]	ICD-8: 286.09, 286.19, 286.29, 286.39, 286.99<br>ICD-10: ICD-10: D66, D67, D680, D681, D682, D689, D691, D693, D694	--
Atrial fibrillation[2]	ICD-8: 427.93, 427.94<br>ICD-10: I48	--

© 2021 Hald SM et al. *JAMA Network Open.*

Heart failure, congestive[2]	ICD-8: 427.09, 427.10, 427.11, 427.19, 428.99, 782.49<br>ICD-10: I110, I42, I50, J819, I130, I132	--
Ischemic heart disease[2]<br>(included as single variable in main analyses; for descriptive purposes divided into components as outlined here)	*1. Myocardial infarct*<br>ICD-8: 410<br>ICD-10: I21, I22, I23<br>*2. Unstable angina*<br>ICD-8: no code available<br>ICD-10: I20.0<br>*3. Other acute ischemic heart disease*<br>ICD-8: 413.09, 413.99<br>ICD-10: I20.1-I20.9, I24.9, I25	--
Venous thromboembolism[2]<br>(DVT or pulmonary embolism)	ICD-8: 450.99, 451x, 671x, 673.09<br>ICD-10: I26, I801,I802, I803, I808, I809	--
Peripheral artery disease[2]	ICD-8: 443.89, 445.00, 445.09, 445.90, 445.99, 440.20, 440.30<br>ICD-10: I702, I739	--
Gastrointestinal bleed[2]<br>(upper, lower, or unspecified)	ICD-8: 45601, 53091, 53098, 53190, 53192, 53195, 53290, 53390, 53490, 53501, 56915<br>ICD-10: K228F, K250, K252, K254, K256, K260, K262, K638B, K264, K266, K270, K272, K274, K276, K280, K282, K284, K286, K290, K298A, K625, K638C, K290-K292, K920, K921, K922	--
Cancer[2]	ICD-8: 140x-207x (173 not included)<br>ICD-10: C00-C97 (C44 not included)	
Disorders or drug use indicative of alcohol misuse[1,2]	ICD-8: 291, 303, 571.09, 571.10, 577.10, 979, 980<br>ICD-10: F10, G312, G621, G721, I426, K292, K70, K860, R780, T51, Z721	N07BB
Chronic obstructive pulmonary disorder	ICD-8: 49000, 49100, 49101, 49103<br>ICD-10: J42, J43, J44	R03 [in subjects aged 45+ years]
**Covariates – use of other drugs[3]**		
Nonsteroidal anti-inflammatory drugs	--	MO1A (including Cox2 inhibitors), excluding M01AX
Selective serotonin reuptake inhibitors	--	N06AB
Statins	--	C10AA

© 2021 Hald SM et al. *JAMA Network Open.*

Hormone replacement therapy	--	G03C, G03DC, G03F
Oral corticosteroids	--	H02AB
**Variables used to describe indication for anticoagulation**		
Knee of hip surgery	Procedure codes: KNFB, KNFC, KNGB, KNGC (Since 1996)	
Mechanical heart valve (independent of atrial fibrillation codes)	Procedure code: KFKD00 KFMD00 KFGE00 KFJF00 (Since 1996)<br>[No specific codes available prior to 1996]	
Valvular atrial fibrillation[4]	ICD-10: I48 & at least one of the following codes<br>i) ICD-10: I050, I052, I080A, I081A, I083A, I342, Z952<br>ii) Procedure code: KFKA, KFKD00, KFKH, KFMD00, KFMH, KFGE00, KFJF00	
Non-valvular atrial fibrillation	ICD-10: I48 and not classified as valvular atrial fibrillation (see above)	

Abbreviations ATC: Anatomical Therapeutic Chemical classification; ICD: International Classification of Diseases
[1]Based on prescriptions presented 1st January 1995 to 1 day prior to index date.
[2]Based on hospital contacts (discharges or outpatient) in period 1977 to 1 day prior to index date.
[3]Based on prescriptions presented in period 1 year to 1 day prior to index date.
[4]Defined in accordance with contraindication for use of DOACs in Denmark, i.e., atrial fibrillation and mechanical heart valves or mitral valve stenosis.

© 2021 Hald SM et al. *JAMA Network Open.*

eTable 2. Characteristics of cases with incident intracerebral hemorrhage and their general population controls, Denmark 2005-2018

	Cases with ICH (n=16,765)	General population controls (n=660,477)	OR (95%CI) adj. for sex, age, and calendar year
Sex			
Men	8,761 (52.3)	343,661 (52.0)	NA[1]
Women	8,004 (47.7)	316,816 (48.0)	NA[1]
Age, median (IQR), years	74.8 (64.8; 82.6)	75.0 (65.0; 82.6)	NA[1]
Age, categories, years			
20-64	4,280 (25.5)	165,519 (25.1)	
65-74	4,190 (25.0)	165,255 (25.0)	
75+	8,295 (49.5)	329,703 (49.9)	
30-day case fatality rate	5,038 (30.1)	NA	
Index year			
2005-2011	8,450 (50.4)	332,574 (50.4)	
2012-2018	8,315 (49.6)	327,903 (49.6)	
Comedication[3]			
Non-aspirin NSAIDs	3,221 (19.2)	120,919 (18.3)	1.06 (1.02; 1.10)
Selective serotonin reuptake inhibitors	2,317 (13.8)	59,194 (9.0)	1.66 (1.58; 1.73)
Statins	4,896 (29.2)	177,460 (26.9)	1.14 (1.10; 1.18)
Oral corticosteroids	1,201 (7.2)	43,992 (6.7)	1.09 (1.02; 1.15)
Hormone replacement therapy (women only)	1,190 (14.9)	50,898 (16.1)	0.92 (0.86; 0.97)
Comorbidity			
Hypertension	10,779 (64.3)	375,363 (56.8)	1.47 (1.42; 1.52)
> 5 years	7,665 (45.7)	270,280 (40.9)	--
< 5 years	3,114 (18.6)	105,083 (15.9)	--
Previous ischemic stroke	2,948 (17.6)	43,813 (6.6)	3.15 (3.02; 3.28)
Diabetes	1,872 (11.2)	69,118 (10.5)	1.08 (1.03; 1.13)
Chronic renal insufficiency	395 (2.4)	8,542 (1.3)	1.86 (1.68; 2.06)
Chronic hepatic disease	290 (1.7)	5,423 (0.8)	2.13 (1.89; 2.40)
Coagulopathy	60 (0.4)	1,047 (0.2)	2.26 (1.74; 2.94)
Atrial fibrillation, any	2,131 (12.7)	48,462 (7.3)	1.90 (1.81; 1.99)
Valvular*	83 (0.5)	1,302 (0.2)	2.54 (2.03; 3.17)
Non-valvular*	2,017 (12.0)	46,351 (7.0)	1.87 (1.78; 1.96)
Mechanical heart valve* [#]	103 (0.6)	1,361 (0.2)	3.00 (2.45; 3.67)
Ischemic heart disease	2,464 (14.7)	91,960 (13.9)	1.07 (1.03; 1.12)
Myocardial infarct*	1,170 (7.0)	43,395 (6.6)	1.07 (1.01; 1.14)
Unstable angina*	375 (2.2)	14,341 (2.2)	1.04 (0.93; 1.15)
Ischemic heart disease, other*	1,969 (11.7)	75,544 (11.4)	1.04 (0.99; 1.09)

© 2021 Hald SM et al. *JAMA Network Open.*

Peripheral artery disease	818 (4.9)	24,098 (3.6)	1.37 (1.27; 1.47)
Venous thromboembolism	750 (4.5)	22,113 (3.3)	1.36 (1.26; 1.47)
Gastrointestinal bleed (upper, lower, or unspecified)	1,272 (7.6)	31,911 (4.8)	1.64 (1.54; 1.74)
Cancer (excl. non-melanoma skin cancer)	2,296 (13.7)	84,230 (12.8)	1.10 (1.05; 1.15)
High alcohol intake	1,360 (8.1)	24,292 (3.7)	2.35 (2.22; 2.49)
Chronic obstructive pulmonary disease	4,443 (26.5)	165,636 (25.1)	1.09 (1.05; 1.12)
Congestive heart failure	710 (4.2)	24,579 (3.7)	1.15 (1.07; 1.25)

Data expressed as numbers (%), unless otherwise stated.

*Defined for descriptive purposes, i.e., information was not entered in multivariable analyses.

[#] Included 36 cases and 484 controls who also suffered from atrial fibrillation (i.e. also included in the 'Atrial fibrillation, Valvular' group).

[1]Not applicable; by design cases and controls were matched on age, sex, and index year.

[2]Data only relevant for cases.

[3]Use of drug between 1 year and 1 day prior to index date.

© 2021 Hald SM et al. *JAMA Network Open.*

eTable 3. Association of antithrombotic drug use with intracerebral hemorrhage stratified by age and sex.

Stratified by sex	Cases, no. (%)	Controls, no. (%)	OR[1] (95% CI)	Adjusted OR[2] (95% CI)	Interaction estimate (95%CI)	Adj.[1] interaction estimate (95%CI)
**Non-use of any antithrombotic drug***						
Women	4,101 (51.2)	210,936 (66.6)	1 (Ref. for women)	1 (Ref. for women)	1 (ref)	1 (ref)
Men	4,253 (48.5)	219,340 (63.8)	1 (Ref. for men)	1 (Ref. for men)	1 (ref)	1 (ref)
**Current use[3] – low-dose aspirin**						
Women	2,254 (28.2)	69,334 (21.9)	1.83 (1.73; 1.93)	1.48 (1.38; 1.58)	1.03 (0.95; 1.11)	1.01 (0.93; 1.09)
Men	2,564 (29.3)	79,908 (23.3)	1.88 (1.78; 1.99)	1.53 (1.43; 1.65)	1 (ref)	1 (ref)
**Current use[3] – clopidogrel**						
Women	470 (5.9)	9,906 (3.1)	2.65 (2.39; 2.94)	1.60 (1.37; 1.87)	1.02 (0.88; 1.18)	0.98 (0.85; 1.14)
Men	566 (6.5)	12,317 (3.6)	2.70 (2.45; 2.98)	1.69 (1.44; 1.97)	1 (ref)	1 (ref)
**Current use[3] – DOAC**						
Women	251 (3.1)	5,623 (1.8)	2.44 (2.11; 2.82)	1.86 (1.55; 2.21)	1.03 (0.83; 1.26)	1.01 (0.82; 1.25)
Men	260 (3.0)	6,151 (1.8)	2.50 (2.16; 2.89)	1.81 (1.51; 2.17)	1 (ref)	1 (ref)
**Current use[3] – VKA**						
Women	833 (10.4)	12,829 (4.0)	3.66 (3.37; 3.97)	2.84 (2.56; 3.14)	0.92 (0.82; 1.03)	0.92 (0.82; 1.03)
Men	1,176 (13.4)	20,446 (5.9)	3.37 (3.13; 3.62)	2.68 (2.44; 2.94)	1 (ref)	1 (ref)
**Stratified by age**	**Cases, no. (%)**	**Controls, no. (%)**	**OR[1] (95% CI)**	**Adjusted OR[2] (95% CI)**	**Interaction estimate (95%CI)**	**Adjusted[1] interaction estimate (95%CI)**
**Non-use of any antithrombotic drug***						
20-64 years	3,282 (76.7)	147,566 (89.2)	1 (Ref. for 20-64)	1 (Ref. for 20-64)	1 (ref)	1 (ref)
65-74 years	2,061 (49.2)	114,096 (69.0)	1 (Ref. for 65-74)	1 (Ref. for 65-74)	1 (ref)	1 (ref)
75+ years	3,011 (36.3)	168,614 (51.1)	1 (Ref. for 75+)	1 (Ref. for 75+)	1 (ref)	1 (ref)
**Current use[3] – low-dose aspirin**						
20-64 years	589 (13.8)	12,269 (7.4)	2.34 (2.13; 2.57)	1.44 (1.26; 1.64)	1.44 (1.30; 1.61)	1.48 (1.33; 1.65)
65-74 years	1,260 (30.1)	34,033 (20.6)	2.08 (1.94; 2.24)	1.59 (1.44; 1.76)	1.28 (1.17; 1.40)	1.34 (1.22; 1.46)

© 2021 Hald SM et al. *JAMA Network Open.*

75+ years	2,969 (35.8)	102,940 (31.2)	1.62 (1.54; 1.71)	1.48 (1.39; 1.58)	1 (ref)	1 (ref)
**Current use[3] – clopidogrel**						
20-64 years	140 (3.3)	1,790 (1.1)	3.59 (2.99; 4.29)	1.41 (1.05; 1.89)	1.60 (1.30; 1.96)	1.45 (1.17; 1.78)
65-74 years	293 (7.0)	4,970 (3.0)	3.37 (2.96; 3.84)	1.82 (1.47; 2.26)	1.50 (1.28; 1.76)	1.42 (1.21; 1.68)
75+ years	603 (7.3)	15,463 (4.7)	2.24 (2.04; 2.47)	1.69 (1.46; 1.95)	1 (ref)	1 (ref)
**Current use[3] – DOAC**						
20-64 years	34 (0.8)	417 (0.3)	3.74 (2.61; 5.37)	2.43 (1.57; 3.77)	1.66 (1.14; 2.44)	1.66 (1.12; 2.45)
65-74 years	113 (2.7)	2,263 (1.4)	2.82 (2.29; 3.46)	1.67 (1.29; 2.17)	1.25 (0.98; 1.59)	1.26 (0.99; 1.61)
75+ years	364 (4.4)	9,094 (2.8)	2.25 (1.99; 2.55)	1.98 (1.70; 2.31)	1 (ref)	1 (ref)
**Current use[3] – VKA**						
20-64 years	193 (4.5)	1,931 (1.2)	4.77 (4.08; 5.58)	2.77 (2.26; 3.41)	1.52 (1.28; 1.81)	1.45 (1.22; 1.73)
65-74 years	484 (11.6)	6,946 (4.2)	4.01 (3.61; 4.46)	2.90 (2.52; 3.34)	1.28 (1.13; 1.45)	1.27 (1.12; 1.45)
75+ years	1,332 (16.1)	24,398 (7.4)	3.13 (2.92; 3.35)	2.82 (2.60; 3.07)	1 (ref)	1 (ref)

[1]Adjusted for calendar year (by design)
[2]Adjusted for all covariates included in main analysis, except the stratifying covariate.
[3]Treatment episode ending 0-30 days before index date

© 2021 Hald SM et al. *JAMA Network Open.*

eTable 4. Use of antiplatelet drugs and risk of intracerebral hemorrhage in Denmark stratified by recency and duration of current use, 2005-2018

	Cases, no. (%) (n=16,765)	Controls, no. (%) (n=660,477)	OR[1] (95% CI)	Adjusted OR[2] (95% CI)
Non-use of any antithrombotic drug*	8,354 (49.8)	430,276 (65.1)	1 (Reference)	1 (Reference)
Low-dose aspirin use[3,4]				
Recency of use[5]				
Current use	4,818 (28.7)	149,242 (22.6)	1.86 (1.78; 1.93)	1.51 (1.44; 1.59)
Recent use	365 (2.2)	10,470 (1.6)	2.00 (1.79; 2.23)	1.50 (1.34; 1.68)
Past use	599 (3.6)	16,234 (2.5)	2.14 (1.96; 2.33)	1.39 (1.26; 1.53)
Past use 91-183[5]	256 (1.5)	7,368 (1.1)	2.00 (1.76; 2.27)	1.37 (1.20; 1.57)
Past use 184-365[5]	343 (2.0)	8,866 (1.3)	2.25 (2.01; 2.52)	1.41 (1.25; 1.59)
Duration of current use				
<1 month	<5	53 (0.0)	2.19 (0.53; 9.06)	1.39 (0.33; 5.85)
≥1 month, ≤3 months	16 (0.1)	236 (0.0)	4.01 (2.41; 6.68)	2.43 (1.44; 4.10)
>3 months, ≤12 months	2,106 (12.6)	55,893 (8.5)	2.14 (2.03; 2.25)	1.69 (1.59; 1.79)
>1 year, ≤3 years	1,804 (10.8)	54,471 (8.2)	1.90 (1.80; 2.00)	1.51 (1.42; 1.60)
>3 years	1,854 (11.1)	65,293 (9.9)	1.65 (1.56; 1.74)	1.29 (1.22; 1.38)
Clopidogrel use[3,4]				
Recency of use[5]				
Current use	1,036 (6.2)	22,223 (3.4)	2.68 (2.50; 2.88)	1.65 (1.47; 1.84)
Recent use	74 (0.4)	1,405 (0.2)	3.05 (2.39; 3.88)	1.82 (1.39; 2.38)
Past use	161 (1.0)	3,552 (0.5)	2.58 (2.19; 3.03)	1.65 (1.34; 2.03)
Past use 91-183[6]	54 (0.3)	1,390 (0.2)	2.20 (1.67; 2.91)	1.37 (1.01; 1.87)
Past use 184-365[6]	107 (0.6)	2,162 (0.3)	2.82 (2.30; 3.44)	1.85 (1.45; 2.36)
Duration of current use				
<1 month	15 (0.1)	270 (0.0)	3.25 (1.90; 5.57)	2.17 (1.23; 3.85)
≥1 month, ≤3 months	50 (0.3)	766 (0.1)	3.59 (2.67; 4.83)	2.46 (1.77; 3.43)
>3 months, ≤12 months	566 (3.4)	10,543 (1.6)	3.04 (2.78; 3.34)	2.08 (1.81; 2.39)
>1 year, ≤3 years	457 (2.7)	10,052 (1.5)	2.62 (2.37; 2.90)	1.69 (1.49; 1.93)
>3 years	183 (1.1)	5,549 (0.8)	1.90 (1.63; 2.21)	1.13 (0.95; 1.34)

*Non-use of any antithrombotic drug is defined as no use of any antiplatelet or anticoagulant in the 12 months preceding index-date

[1]Adjusted for age, sex, and calendar period (year) by design

[2]Adjusted for age, sex, and calendar period (by design) and the following, based on register data: hypertension, previous ischemic stroke, diabetes, chronic renal insufficiency, chronic hepatic disease, coagulopathy, heart failure, ischemic heart disease, peripheral artery disease, cancer, high alcohol consumption, chronic obstructive pulmonary disease, use of oral anticoagulants, low-dose aspirin, clopidogrel, other adenosine diphosphate inhibitors (ticagrelor or prasugrel), statins, nonsteroidal anti-inflammatory drugs, selective serotonin reuptake inhibitors, hormone replacement therapy, or oral corticosteroid drugs.

[3]Within last 12 months before index-date.

[4]Concurrent use or previous use (within 12 months before index date) of other antithrombotic drugs included.

[5]Based on the most recent treatment episode prior to the index date (date of diagnosis for cases and date of selection for controls), exposure was divided into current use (treatment episode ending 0-30 days before index date), recent use (31-90 days before index date), and past use (91-365 days before index date).

[6]Post hoc analysis with past use classified into 91-183 days and 184-365 days.

© 2021 Hald SM et al. *JAMA Network Open.*

eTable 5. Use of anticoagulant drugs and risk of intracerebral hemorrhage in Denmark stratified by recency and duration of current use, 2005-2018.

	Cases, no. (%) (n=16,765)	Controls, no. (%) (n=660,477)	OR[1] (95% CI)	Adjusted OR[2] (95% CI)
Non-use of any antithrombotic drug*	8,354 (49.8)	430,276 (65.1)	1 (Reference)	1 (Reference)
Direct oral anticoagulant use[3,4]				
Recency of use[5]				
Current use	511 (3.0)	11,774 (1.8)	2.47 (2.23; 2.73)	1.83 (1.61; 2.07)
Recent use	18 (0.1)	682 (0.1)	1.42 (0.88; 2.28)	0.97 (0.59; 1.60)
Past use – any	42 (0.3)	1,419 (0.2)	1.65 (1.20; 2.25)	1.18 (0.84; 1.66)
Past use 91-183[6]	14 (0.1)	583 (0.1)	1.36 (0.80; 2.34)	1.04 (0.59; 1.82)
Past use 184-365[6]	28 (0.2)	836 (0.1)	1.84 (1.25; 2.70)	1.27 (0.84; 1.92)
Duration of current use				
<1 month	16 (0.1)	481 (0.1)	1.81 (1.09; 3.01)	1.48 (0.88; 2.51)
≥1 month, ≤3 months	60 (0.4)	1,629 (0.2)	2.12 (1.62; 2.76)	1.54 (1.14; 2.08)
>3 months, ≤12 months	233 (1.4)	4,786 (0.7)	2.74 (2.37; 3.15)	2.16 (1.80; 2.59)
>1 year, ≤3 years	213 (1.3)	4,944 (0.7)	2.42 (2.09; 2.81)	1.85 (1.57; 2.17)
>3 years	49 (0.3)	2,035 (0.3)	1.34 (1.00; 1.80)	1.01 (0.75; 1.36)
Vitamin K antagonist use[3,4]				
Recency of use[5]				
Current use	2,009 (12.0)	33,275 (5.0)	3.49 (3.31; 3.69)	2.76 (2.58; 2.96)
Recent use	193 (1.2)	3,145 (0.5)	3.53 (3.03; 4.11)	2.69 (2.29; 3.16)
Past use – any	150 (0.9)	4,070 (0.6)	2.11 (1.78; 2.50)	1.51 (1.25; 1.82)
Past use 91-183[6]	88 (0.5)	1,852 (0.3)	2.72 (2.18; 3.39)	1.95 (1.54; 2.47)
Past use 184-365[6]	62 (0.4)	2,218 (0.3)	1.60 (1.24; 2.07)	1.13 (0.86; 1.49)
Duration of current use				
<1 month	<5	56 (0.0)	1.91 (0.46; 7.97)	1.51 (0.36; 6.35)
≥1 month, ≤3 months	368 (2.2)	5,129 (0.8)	4.21 (3.76; 4.72)	3.18 (2.80; 3.60)
>3 months, ≤12 months	618 (3.7)	10,582 (1.6)	3.36 (3.07; 3.66)	2.62 (2.37; 2.90)
>1 year, ≤3 years	622 (3.7)	10,301 (1.6)	3.44 (3.15; 3.75)	2.81 (2.55; 3.09)
>3 years	742 (4.4)	14,422 (2.2)	3.00 (2.77; 3.25)	2.48 (2.27; 2.71)

*Non-use of any antithrombotic drug is defined as no use of any antiplatelet or anticoagulant in the 12 months preceding index-date

[1]Adjusted for age, sex, and calendar period (year) by design

[2]Adjusted for age, sex, and calendar period (by design) and the following, based on register data: hypertension, previous ischemic stroke, diabetes, chronic renal insufficiency, chronic hepatic disease, coagulopathy, heart failure, ischemic heart disease, peripheral artery disease, cancer, high alcohol consumption, chronic obstructive pulmonary disease, use of oral anticoagulants, low-dose aspirin, clopidogrel, other adenosine diphosphate inhibitors (ticagrelor or prasugrel), statins, nonsteroidal anti-inflammatory drugs, selective serotonin reuptake inhibitors, hormone replacement therapy, or oral corticosteroid drugs.

[3]Within last 12 months before index-date.

[4]Concurrent use or previous use (within 12 months before index date) of other antithrombotic drugs included.

[5]Based on the most recent treatment episode prior to the index date (date of diagnosis for cases and date of selection for controls), exposure was divided into current use (treatment episode ending 0-30 days before index date), recent use (31-90 days before index date), and past use (91-365 days before index date).

[6]Post hoc analysis with past use classified into 91-183 days and 184-365 days.

© 2021 Hald SM et al. *JAMA Network Open.*

eTable 6. Current use of anticoagulants in patients with atrial fibrillation and venous thromboembolism and risk of intracerebral hemorrhage in Denmark, 2005-2018

	Cases, no. (%)	Controls, no. (%)	OR[1] (95% CI)	Adj. OR[2] (95%CI)
**Atrial fibrillation**[3]	(n=2,131)	(n=48,462)		
Non-use of any antithrombotic drug*	137 (6.4)	5,909 (12.2)	1 (Reference)	1 (Reference)
Current use of oral anticoagulant[4]				
All current use[5]				
DOAC	307 (14.4)	7,609 (15.7)	1.74 (1.42; 2.13)	1.64 (1.32; 2.05)
Dabigatran	54 (2.5)	2,820 (5.8)	0.83 (0.60; 1.14)	0.80 (0.56; 1.13)
Rivaroxaban	153 (7.2)	2,263 (4.7)	2.92 (2.30; 3.69)	2.79 (2.16; 3.61)
Apixaban	100 (4.7)	2,514 (5.2)	1.72 (1.32; 2.23)	1.57 (1.17; 2.11)
Edoxaban	<5	59 (0.1)	NR	NR
VKA	1,157 (54.3)	20,063 (41.4)	2.49 (2.08; 2.98)	2.51 (2.08; 3.03)
Warfarin	1,136 (53.3)	19,533 (40.3)	2.51 (2.10; 3.00)	2.53 (2.10; 3.06)
Single use only[6]				
DOAC	199 (9.3)	5,304 (10.9)	1.62 (1.30; 2.02)	1.57 (1.24; 1.99)
Dabigatran	37 (1.7)	2,037 (4.2)	0.78 (0.54; 1.13)	0.74 (0.50; 1.10)
Rivaroxaban	93 (4.4)	1,445 (3.0)	2.78 (2.12; 3.63)	2.66 (2.00; 3.55)
Apixaban	54 (2.5)	1,581 (3.3)	1.47 (1.07; 2.03)	1.43 (1.01; 2.02)
Edoxaban	<5	14 (0.0)	NR	NR
VKA	714 (33.5)	14,263 (29.4)	2.16 (1.79; 2.60)	2.29 (1.89; 2.78)
Warfarin	697 (32.7)	13,806 (28.5)	2.18 (1.81; 2.62)	2.31 (1.91; 2.81)
**Deep venous thrombosis/pulmonary embolism**[3,7]	(n=750)	(n=22,113)		
Non-use of any antithrombotic drug*	192 (25.6)	7,741 (35.0)	1 (Reference)	1 (Reference)
Current use of oral anticoagulant[4]				
All current use[5]				
DOAC	68 (9.1)	1,311 (5.9)	2.09 (1.58; 2.77)	1.99 (1.46; 2.71)
Dabigatran	<5	217 (1.0)	NR	NR
Rivaroxaban	47 (6.3)	762 (3.4)	2.49 (1.79; 3.45)	2.36 (1.66; 3.36)
Apixaban	18 (2.4)	327 (1.5)	2.22 (1.35; 3.64)	1.89 (1.11; 3.22)
Edoxaban	<5	10 (0.0)	NR	NR
VKA	263 (35.1)	4,814 (21.8)	2.20 (1.82; 2.66)	2.21 (1.80; 2.71)
Warfarin	254 (33.9)	4,660 (21.1)	2.20 (1.82; 2.66)	2.21 (1.80; 2.71)
Single current use only[6]				
DOAC	47 (6.3)	879 (4.0)	2.16 (1.56; 2.99)	2.06 (1.46; 2.90)
Dabigatran	<5	141 (0.6)	NR	NR

© 2021 Hald SM et al. *JAMA Network Open.*

Rivaroxaban	33 (4.4)	521 (2.4)	2.55 (1.75; 3.73)	2.47 (1.67; 3.66)
Apixaban	9 (1.2)	187 (0.8)	1.94 (0.98; 3.85)	1.54 (0.75; 3.14)
Edoxaban	<5	<5	NR	NR
VKA	175 (23.3)	3,675 (16.6)	1.92 (1.56; 2.37)	2.02 (1.62; 2.52)
Warfarin	166 (22.1)	3,530 (16.0)	1.90 (1.53; 2.34)	2.01 (1.61; 2.51)

NR: Not reported due to sparse data in accordance with regulations of Danish Health Data Authority.

*Non-use of any antithrombotic drug is defined as no use of any antiplatelet or anticoagulant in the 12 months preceding index-date

[1]Adjusted for age, sex, and calendar period (year) by design.

[2]Adjusted for age, sex, and calendar period (by design) and the following, based on register data: hypertension, previous ischemic stroke, diabetes, chronic renal insufficiency, chronic hepatic disease, coagulopathy, heart failure, ischemic heart disease, peripheral artery disease, cancer, high alcohol consumption, chronic obstructive pulmonary disease, use of oral anticoagulants, low-dose aspirin, clopidogrel, other adenosine diphosphate inhibitors (ticagrelor or prasugrel), statins, nonsteroidal anti-inflammatory drugs, selective serotonin reuptake inhibitors, hormone replacement therapy, or oral corticosteroid drugs.

[3]Cases and controls restricted to patients with the diagnosis at any time prior to index date.

[4]Use at index date.

[5]Concurrent use or previous use (within 12 months before index date) of other antithrombotic drugs included.

[6]Concurrent use or previous use (within 12 months before index date) of other antithrombotic drugs not included.

[7]Cases with both atrial fibrillation and deep venous thrombosis / pulmonary embolism diagnoses were classified as atrial fibrillation.

© 2021 Hald SM et al. *JAMA Network Open.*

eTable 7. Current use of antithrombotic drugs and risk of intracerebral hemorrhage in Denmark, 2014-2018

	**Cases, no. (%) (n=6,002)**	**Controls, no. (%) (n=236,742)**	**OR[1] (95% CI)**	**Adjusted OR[2] (95% CI)**
Non-use of any antithrombotic drug*	2,950 (49.2)	153,014 (64.6)	1 (Reference)	1 (Reference)
Current use of antiplatelet drug				
Low-dose aspirin use[3,4]	1,204 (20.1)	41,900 (17.7)	1.67 (1.55; 1.79)	1.47 (1.34; 1.61)
Clopidogrel use[3,4]	665 (11.1)	13,879 (5.9)	2.87 (2.62; 3.15)	2.11 (1.84; 2.43)
Current use of anticoagulant drug				
Direct oral anticoagulant use[3,4]	476 (7.9)	10,769 (4.5)	2.53 (2.27; 2.82)	2.03 (1.78; 2.32)
Dabigatran	58 (1.0)	3,138 (1.3)	1.06 (0.81; 1.38)	0.86 (0.63; 1.18)
Rivaroxaban	252 (4.2)	3,770 (1.6)	3.75 (3.25; 4.33)	3.15 (2.63; 3.77)
Apixaban	164 (2.7)	3,831 (1.6)	2.47 (2.08; 2.93)	1.79 (1.42; 2.25)
Edoxaban	5 (0.1)	96 (0.0)	3.04 (1.19; 7.78)	4.57 (1.27; 16.48)
Vitamin K antagonist use[3,4]	643 (10.7)	12,086 (5.1)	3.14 (2.85; 3.45)	2.64 (2.36; 2.96)
Warfarin	634 (10.6)	11,909 (5.0)	3.14 (2.85; 3.46)	2.63 (2.34; 2.96)

*Non-use of any antithrombotic drug is defined as no use of any antiplatelet or anticoagulant in the 12 months preceding index-date

[1]Adjusted for age, sex, and calendar period (year) by design

[2]Adjusted for age, sex, and calendar period (by design) and the following, based on register data: hypertension, previous ischemic stroke, diabetes, chronic renal insufficiency, chronic hepatic disease, coagulopathy, heart failure, ischemic heart disease, peripheral artery disease, cancer, high alcohol consumption, chronic obstructive pulmonary disease, use of oral anticoagulants, low-dose aspirin, clopidogrel, other adenosine diphosphate inhibitors (ticagrelor or prasugrel), statins, nonsteroidal anti-inflammatory drugs, selective serotonin reuptake inhibitors, hormone replacement therapy, or oral corticosteroid drugs. In drug-specific analyses of anticoagulants use of oral anticoagulants we adjusted with individual variables for use of dabigatran, rivaroxaban, apixaban, edoxaban, or warfarin, respectively.

[3]Within last 12 months before index-date.

[4]Concurrent use or previous use (within 12 months before index date) of other antithrombotic drugs included.

© 2021 Hald SM et al. *JAMA Network Open.*

eTable 8. Duration of current naive use of specific anticoagulants and risk of intracerebral hemorrhage in Denmark, 2014-2018

**Anticoagulant type**	**Cases, no. (%) (n=6,002)**	**Controls, no. (%) (n=236,742)**	**OR[1] (95% CI)**	**Adjusted OR[2] (95% CI)**
Non-use of any antithrombotic drug*	2,924 (48.7)	151,628 (64.0)	1 (Reference)	1 (Reference)
Current naive# oral anticoagulant use[3,4] – duration				
Dabigatran				
< 3 months	<5	153 (0.1)	1.29 (0.40; 4.14)	1.02 (0.31; 3.31)
3-12 months	9 (0.1)	403 (0.2)	1.25 (0.64; 2.45)	1.05 (0.53; 2.09)
>1 year	28 (0.5)	1,953 (0.8)	0.81 (0.56; 1.19)	0.69 (0.46; 1.02)
Rivaroxaban				
< 3 months	8 (0.1)	138 (0.1)	3.15 (1.50; 6.60)	2.91 (1.37; 6.18)
3-12 months	79 (1.3)	1,115 (0.5)	4.07 (3.18; 5.19)	3.46 (2.68; 4.46)
>1 year	88 (1.5)	1,414 (0.6)	3.57 (2.83; 4.51)	3.02 (2.36; 3.85)
Apixaban				
< 3 months	15 (0.2)	315 (0.1)	2.72 (1.58; 4.69)	1.97 (1.12; 3.46)
3-12 months	49 (0.8)	932 (0.4)	3.00 (2.21; 4.07)	2.47 (1.80; 3.39)
>1 year	57 (0.9)	1,407 (0.6)	2.36 (1.78; 3.13)	1.78 (1.32; 2.40)
Warfarin				
< 3 months	77 (1.3)	990 (0.4)	4.79 (3.74; 6.15)	4.05 (3.12; 5.25)
3-12 months	115 (1.9)	2,039 (0.9)	3.33 (2.72; 4.06)	2.80 (2.27; 3.46)
>1 year	405 (6.7)	8,199 (3.5)	2.92 (2.60; 3.28)	2.62 (2.30; 2.97)

Naive use: No use of other anticoagulant at any point in time before index date.

*Non-use of any antithrombotic drug is defined as no use of any antiplatelet or anticoagulant in the 12 months preceding index-date

[1]Adjusted for age, sex, and calendar period (year) by design

[2]Adjusted for age, sex, and calendar period (by design) and the following, based on register data: hypertension, previous ischemic stroke, diabetes, chronic renal insufficiency, chronic hepatic disease, coagulopathy, heart failure, ischemic heart disease, peripheral artery disease, cancer, high alcohol consumption, chronic obstructive pulmonary disease, use of oral anticoagulants, low-dose aspirin, clopidogrel, other adenosine diphosphate inhibitors (ticagrelor or prasugrel), statins, nonsteroidal anti-inflammatory drugs, selective serotonin reuptake inhibitors, hormone replacement therapy, or oral corticosteroid drugs.

[3]Based on the most recent treatment episode prior to the index date (date of diagnosis for cases and date of selection for controls), current use was defined as treatment episode ending 0-30 days before index date.

[4]Concurrent use or previous use (within 12 months before index date) of antiplatelet drugs included.

© 2021 Hald SM et al. *JAMA Network Open.*

eTable 9. Current use of DOAC vs current use of VKA and risk of intracerebral hemorrhage in Denmark

Entire study period 2005-2018	Cases, no. (%) (n=16,765)	Controls, no. (%) (n=660,477)	OR[1] (95% CI)	Adjusted OR[2] (95% CI)
**Current use[3,4] – main analysis[5]**				
DOAC	500 (3.0)	11,526 (1.7)	0.72 (0.65; 0.79)	0.73 (0.66; 0.81)
VKA	1,998 (11.9)	33,027 (5.0)	1 (ref)	1 (ref)
**Current use[3,4] – monotherapy[6]**				
DOAC	357 (2.1)	8,792 (1.3)	0.76 (0.67; 0.85)	0.73 (0.64; 0.82)
VKA	1,267 (7.6)	23,579 (3.6)	1 (ref)	1 (ref)
**Current use[3,4] – new use[7]**				
DOAC	409 (2.4)	9,277 (1.4)	0.71 (0.63; 0.79)	0.72 (0.63; 0.81)
VKA	1,030 (6.1)	16,488 (2.5)	1 (ref)	1 (ref)
**Current use[3] – naive use[8]**				
DOAC	400 (2.4)	9,220 (1.4)	0.72 (0.64; 0.80)	0.72 (0.65; 0.81)
VKA	1,967 (11.7)	32,462 (4.9)	1 (ref)	1 (ref)
**Restricted study period 2014-2018**	**Cases, no. (%) (n=6,002)**	**Controls, no. (%) (n=236,742)**	**OR[1] (95% CI)**	**Adjusted OR[2] (95% CI)**
**Current use[3,4] – main analysis[5]**				
DOAC	467 (7.8)	10,573 (4.5)	0.83 (0.73; 0.94)	0.78 (0.65; 0.89)
VKA	634 (10.6)	11,890 (5.0)	1 (ref)	1 (ref)
**Current use[3,4] – monotherapy[6]**				
DOAC	343 (5.7)	8,267 (3.5)	0.83 (0.72; 0.96)	0.79 (0.69; 0.92)
VKA	482 (8.0)	9,679 (4.1)	1 (ref)	1 (ref)
**Current use[3,4] – new use[7]**				
DOAC	387 (6.3)	8,392 (3.5)	0.84 (0.72; 0.98)	0.79 (0.68; 0.93)
VKA	305 (5.1)	5,666 (2.4)	1 (ref)	1 (ref)
**Current use[3,4] – naive use[8]**				
DOAC	379 (6.3)	8,574 (3.6)	0.83 (0.73; 0.95)	0.78 (0.68; 0.89)
VKA	609 (10.1)	11,432 (4.8)	1 (ref)	1 (ref)

Patients recorded as current users of both DOAC and VKA (e.g., 11 cases and 248 controls in main analysis of entire study period) were not included in this analysis.

© 2021 Hald SM et al. *JAMA Network Open.*

[1]Adjusted for age, sex, and calendar period (year)

[2]Adjusted for age, sex, and calendar period and the following, based on register data: hypertension, previous ischemic stroke, diabetes, chronic renal insufficiency, chronic hepatic disease, coagulopathy, heart failure, ischemic heart disease, peripheral artery disease, cancer, high alcohol consumption, chronic obstructive pulmonary disease, use of oral anticoagulants, low-dose aspirin, clopidogrel, other adenosine diphosphate inhibitors (ticagrelor or prasugrel), statins, nonsteroidal anti-inflammatory drugs, selective serotonin reuptake inhibitors, hormone replacement therapy, or oral corticosteroid drugs.

[3]Based on the most recent treatment episode prior to the index date (date of diagnosis for cases and date of selection for controls), current use was defined as treatment episode ending 0-30 days before index date.

[4]Concurrent use or previous use (within 12 months before index date) of antiplatelet drugs included.

[5]Previous use of other anticoagulants included.

[6]No use of any other anticoagulant or antiplatelet drug (i.e. any antithrombotic) within 12 months of index date

[7]Only a single episode of treatment within the drug class/specific drug recorded before index date and no recorded use in 1995-1996 (Prescription Registry operational since 1995).

[8]Never use of any other anticoagulant before index date.

© 2021 Hald SM et al. *JAMA Network Open.*

eTable 10. Dose of current use of specific anticoagulants and risk of intracerebral hemorrhage in Denmark, **2014-2018**

**Anticoagulant type**	**Cases, no. (%) (n=6,002)**	**Controls, no. (%) (n=236,742)**	**OR[1] (95% CI)**	**Adjusted OR[2] (95% CI)**
Non-use of any antithrombotic drug*	2,950 (49.2)	153,014 (64.6)	1 (Reference)	1 (Reference)
Current oral anticoagulant use[3,4] – Dose[#]				
Any type of DOAC[5]				
Reduced dose vs non-use	194 (3.2)	5,269 (2.2)	1.91 (1.65; 2.21)	1.52 (1.28; 1.80)
Standard dose vs non-use	277 (4.6)	5,406 (2.3)	2.66 (2.34; 3.01)	2.21 (1.91; 2.56)
Standard dose vs reduced dose	NA	NA	1.39 (1.15; 1.68)	1.41 (1.16; 1.70)
Dabigatran				
Reduced dose (≤220 mg) vs non-use	37 (0.6)	2,070 (0.9)	0.93 (0.67; 1.29)	0.77 (0.54; 1.10)
Standard dose (300 mg) vs. non-use	21 (0.3)	1,068 (0.5)	1.02 (0.66; 1.57)	0.90 (0.57; 1.42)
Standard dose vs reduced dose	NA	NA	1.10 (0.64; 1.89)	1.17 (0.67; 2.04)
Rivaroxaban				
Reduced dose (≤15 mg) vs non-use	82 (1.4)	1,341 (0.6)	3.17 (2.53; 3.98)	2.49 (1.94; 3.21)
Standard dose (20 mg) vs. non-use	170 (2.8)	2,429 (1.0)	3.63 (3.09; 4.26)	3.07 (2.56; 3.69)
Standard dose vs reduced dose	NA	NA	1.14 (0.87; 1.50)	1.17 (0.88; 1.56)
Apixaban				
Reduced dose (≤5 mg)	77 (1.3)	1,903 (0.8)	2.10 (1.67; 2.64)	1.48 (1.13; 1.94)
Standard dose (10 mg)	87 (1.4)	1,928 (0.8)	2.34 (1.88; 2.91)	1.69 (1.32; 2.18)
Standard dose vs reduced dose	NA	NA	1.12 (0.82; 1.53)	1.02 (0.73; 1.41)

#Classified based on dose of last prescription presented before index date.

*Non-use of any antithrombotic drug is defined as no use of any antiplatelet or anticoagulant in the 12 months preceding index-date

[1]Adjusted for age, sex, and calendar period (year) by design

[2]Adjusted for age, sex, and calendar period (by design) and the following, based on register data: hypertension, previous ischemic stroke, diabetes, chronic renal insufficiency, chronic hepatic disease, coagulopathy, heart failure, ischemic heart disease, peripheral artery disease, cancer, high alcohol consumption, chronic obstructive pulmonary disease, use of oral anticoagulants, low-dose aspirin, clopidogrel, other adenosine diphosphate inhibitors (ticagrelor or prasugrel), statins, nonsteroidal anti-inflammatory drugs, selective serotonin reuptake inhibitors, hormone replacement therapy, or oral corticosteroid drugs.

[3]Based on the most recent treatment episode prior to the index date (date of diagnosis for cases and date of selection for controls), current use was defined as treatment episode ending 0-30 days before index date.

[4]Concurrent use or previous use (within 12 months before index date) of other antithrombotic drugs included.

[5]Current users of more than one DOAC (<5 cases and 31 controls), e.g., due to switching, only contributed once in analyses of DOAC as a class of drugs.

© 2021 Hald SM et al. *JAMA Network Open.*

eTable 11. Odds ratios for association of antithrombotic drug use in models with and without adjustment for socioeconomic status, Denmark 2005-2016

Cases ascertained through Danish Stroke Registry as in main analyses.

			**Adjusted OR (95% CI)**	
**Use of antithrombotic drug**	**Cases, no. (%) (n=14,084)**	**Controls, no. (%) (n=556,657)**	**SES[1,2] not included in model**	**SES[1,3] included in model**
Non-use of any antithrombotic drug*	7,101 (50.4)	367,606 (66.0)	1 (reference)	1 (reference)
Current use[4,5]				
Low-dose aspirin	4,285 (30.4)	129,903 (23.3)	1.48 (1.40-1.56)	1.48 (1.40-1.56)
Clopidogrel	726 (5.2)	14,665 (2.6)	1.53 (1.34-1.75)	1.54 (1.34-1.76)
VKA	1,760 (12.5)	27,801 (5.0)	2.72 (2.53-2.93)	2.75 (2.55-2.97)
DOAC	219 (1.6)	5,308 (1,0)	1.42 (1.17-1.72)	1.45 (1.19-1.77)

*Non-use of any antithrombotic drug is defined as no use of any antiplatelet or anticoagulant in the 12 months preceding index-date

[1]Socioeconomic status

[2]Adjusted for age, sex, and calendar period (by design) and the following, based on register data: hypertension, previous ischemic stroke, diabetes, chronic renal insufficiency, chronic hepatic disease, coagulopathy, heart failure, ischemic heart disease, peripheral artery disease, cancer, high alcohol consumption, chronic obstructive pulmonary disease, use of oral anticoagulants, low-dose aspirin, clopidogrel, other adenosine diphosphate inhibitors (ticagrelor or prasugrel), statins, nonsteroidal anti-inflammatory drugs, selective serotonin reuptake inhibitors, hormone replacement therapy, or oral corticosteroid drugs. Not adjusted for socioeconomic status

[3]Adjusted for all of above and, in addition, for socioeconomic status by the inclusion of separate covariates for highest educational level and income ascertained the year before ICH onset.

[4]Based on the most recent treatment episode prior to the index date (date of diagnosis for cases and date of selection for controls), current use was defined as treatment episode ending 0-30 days before index date.

[5]Concurrent use or previous use (within 12 months before index date) of antiplatelet drugs included.

© 2021 Hald SM et al. *JAMA Network Open.*

eTable 12. Annual number of cases and incidence rate of intracerebral hemorrhage per 100,000 person-years in Denmark, 2005-2018

	WOMEN								
	Age 20-64		Age 65-74		Age 75+		All		
	Cases	Incidence rate	Cases	Incidence rate	Cases	Incidence rate	Cases	Incidence rate	Standardised incidence rate
2005	105	6.5 (5.3; 7.8)	150	65.7 (55.6; 77.1)	389	164 (148; 182)	644	30.8 (28.5; 33.3)	32.0 (29.7; 34.5)
2006	111	6.8 (5.6; 8.2)	120	51.6 (42.8; 61.7)	353	149 (134; 166)	584	27.9 (25.7; 30.3)	28.7 (26.5; 31.1)
2007	117	7.2 (6.0; 8.6)	129	54.3 (45.4; 64.6)	330	140 (125; 156)	576	27.4 (25.3; 29.8)	28.3 (26.1; 30.6)
2008	85	5.2 (4.2; 6.5)	114	46.5 (38.3; 55.8)	331	140 (126; 157)	530	25.1 (23.1; 27.4)	25.8 (23.8; 28.1)
2009	129	7.9 (6.6; 9.4)	141	55.2 (46.5; 65.1)	344	146 (131; 162)	614	29.0 (26.7; 31.3)	29.7 (27.4; 32.1)
2010	120	7.4 (6.1; 8.8)	124	46.4 (38.6; 55.3)	345	146 (131; 163)	589	27.6 (25.4; 30.0)	28.1 (25.9; 30.4)
2011	120	7.4 (6.1; 8.8)	111	39.6 (32.6; 47.7)	320	135 (121; 151)	551	25.7 (23.6; 27.9)	25.9 (23.9; 28.2)
2012	91	5.6 (4.5; 6.9)	79	26.9 (21.3; 33.5)	314	131 (117; 147)	484	22.4 (20.5; 24.5)	22.4 (20.5; 24.5)
2013	115	7.1 (5.8; 8.5)	134	43.9 (36.8; 51.9)	329	136 (122; 151)	578	26.6 (24.5; 28.9)	26.3 (24.3; 28.6)
2014	107	6.6 (5.4; 7.9)	137	43.6 (36.6; 51.5)	311	126 (113; 141)	555	25.4 (23.3; 27.6)	24.9 (22.9; 27.1)
2015	104	6.4 (5.2; 7.7)	127	39.5 (32.9; 47.0)	331	132 (118; 147)	562	25.5 (23.4; 27.7)	24.8 (22.8; 27.0)
2016	116	7.0 (5.8; 8.5)	152	46.4 (39.3; 54.4)	339	132 (119; 147)	607	27.2 (25.1; 29.5)	26.3 (24.2; 28.5)
2017	96	5.8 (4.7; 7.1)	130	39.2 (32.8; 46.6)	382	146 (132; 161)	608	27.0 (24.9; 29.3)	25.9 (23.8; 28.1)
2018	98	5.9 (4.8; 7.2)	115	34.5 (28.5; 41.4)	309	114 (102; 128)	522	23.0 (21.1; 25.1)	21.8 (19.9; 23.9)
	MEN								
	Age 20-64		Age 65-74		Age 75+		All		
	Cases	Incidence rate	Cases	Incidence rate	Cases	Incidence rate	Cases	Incidence rate	Standardised incidence rate
2005	192	11.6 (10.0; 13.4)	153	74.8 (63.4; 87.6)	257	180 (159; 204)	602	30.1 (27.8; 32.6)	32.9 (30.5; 35.5)
2006	216	13.1 (11.4; 15.0)	156	74.3 (63.1; 86.9)	247	172 (151; 194)	619	30.9 (28.5; 33.4)	33.4 (31.0; 35.9)
2007	218	13.2 (11.5; 15.1)	159	73.7 (62.7; 86.0)	247	170 (149; 192)	624	31.0 (28.6; 33.6)	33.2 (30.8; 35.8)
2008	214	13.0 (11.3; 14.8)	145	64.4 (54.3; 75.8)	251	171 (150; 193)	610	30.1 (27.8; 32.6)	31.9 (29.5; 34.4)
2009	221	13.4 (11.7; 15.2)	184	78.1 (67.2; 90.2)	258	173 (153; 196)	663	32.5 (30.1; 35.1)	34.2 (31.8; 36.8)
2010	194	11.8 (10.2; 13.5)	175	70.5 (60.4; 81.7)	268	177 (156; 199)	637	31.1 (28.7; 33.6)	32.2 (29.9; 34.7)
2011	194	11.8 (10.2; 13.6)	163	62.2 (53.0; 72.5)	250	162 (142; 183)	607	29.4 (27.1; 31.8)	30.0 (27.7; 32.4)
2012	208	12.7 (11.0; 14.5)	190	68.7 (59.3; 79.2)	233	147 (129; 167)	631	30.4 (28.1; 32.8)	30.4 (28.1; 32.8)
2013	182	11.1 (9.5; 12.8)	183	63.4 (54.6; 73.3)	255	156 (137; 176)	620	29.6 (27.3; 32.0)	29.1 (26.9; 31.5)
2014	176	10.7 (9.2; 12.4)	158	53.2 (45.2; 62.1)	233	138 (121; 157)	567	26.8 (24.7; 29.1)	26.1 (24.0; 28.4)
2015	195	11.8 (10.2; 13.5)	176	57.9 (49.7; 67.2)	265	151 (134; 171)	636	29.8 (27.5; 32.2)	28.6 (26.4; 31.0)
2016	217	13.0 (11.3; 14.8)	221	71.5 (62.4; 81.6)	246	135 (119; 154)	684	31.6 (29.3; 34.0)	30.1 (27.8; 32.6)

© 2021 Hald SM et al. *JAMA Network Open.*

2017	175	10.4 (8.9; 12.0)	186	59.4 (51.2; 68.6)	296	157 (140; 176)	657	30.0 (27.7; 32.4)	28.1 (25.9; 30.5
2018	164	9.7 (8.3; 11.3)	178	56.6 (48.6; 65.6)	262	132 (117; 149)	604	27.4 (25.2; 29.7)	25.3 (23.2; 27.6)
	**MEN & WOMEN**								
	**Age 20-64**		**Age 65-74**		**Age 75+**		**All**		
	**Cases**	**Incidence rate**	**Cases**	**Incidence rate**	**Cases**	**Incidence rate**	**Cases**	**Incidence rate**	**Standardised incidence rate**
2005	297	9.1 (8.1; 10.2)	303	70.0 (62.3; 78.3)	646	170 (157; 184)	1246	30.5 (28.8; 32.2)	32.4 (30.7; 34.2)
2006	327	10.0 (8.9; 11.1)	276	62.4 (55.3; 70.2)	600	158 (145; 171)	1203	29.4 (27.7; 31.1)	30.9 (29.3; 32.6)
2007	335	10.2 (9.2; 11.4)	288	63.5 (56.4; 71.3)	577	151 (139; 164)	1200	29.2 (27.6; 30.9)	30.7 (29.0; 32.4)
2008	299	9.1 (8.1; 10.2)	259	55.1 (48.6; 62.2)	582	152 (140; 165)	1140	27.6 (26.0; 29.2)	28.7 (27.2; 30.4)
2009	350	10.7 (9.6; 11.8)	325	66.2 (59.2; 73.8)	602	157 (144; 170)	1277	30.7 (29.0; 32.4)	31.8 (30.2; 33.6)
2010	314	9.6 (8.5; 10.7)	299	58.0 (51.6; 64.9)	613	158 (146; 171)	1226	29.3 (27.7; 31.0)	30.1 (28.5; 31.8)
2011	314	9.6 (8.6; 10.7)	274	50.5 (44.7; 56.8)	570	146 (134; 158)	1158	27.5 (26.0; 29.1)	27.9 (26.3; 29.5)
2012	299	9.2 (8.1; 10.3)	269	47.2 (41.7; 53.1)	547	138 (126; 150)	1115	26.3 (24.8; 27.9)	26.3 (24.8; 27.9)
2013	297	9.1 (8.1; 10.2)	317	53.4 (47.7; 59.6)	584	144 (132; 156)	1198	28.1 (26.5; 29.7)	27.7 (26.2; 29.4)
2014	283	8.6 (7.7; 9.7)	295	48.2 (42.9; 54.1)	544	131 (120; 143)	1122	26.1 (24.6; 27.7)	25.5 (24.0; 27.0)
2015	299	9.1 (8.1; 10.2)	303	48.5 (43.2; 54.2)	596	140 (129; 152)	1198	27.6 (26.0; 29.2)	26.7 (25.2; 28.3)
2016	333	10.0 (9.0; 11.2)	373	58.6 (52.8; 64.8)	585	134 (123; 145)	1291	29.4 (27.8; 31.0)	28.2 (26.6; 29.8)
2017	271	8.1 (7.2; 9.1)	316	49.0 (43.8; 54.7)	678	150 (139; 162)	1265	28.5 (27.0; 30.1)	27.0 (25.5; 28.6)
2018	262	7.8 (6.9; 8.8)	293	45.2 (40.2; 50.7)	571	122 (112; 132)	1126	25.2 (23.7; 26.7)	23.6 (22.1; 25.2)

© 2021 Hald SM et al. *JAMA Network Open.*

eTable 13. Incidence rate ratio of intracerebral hemorrhage in 2005–2011 versus 2012–2018 using Stroke Registry and Patient Registry – for entire Danish population (5.8 million) and limited to population of Western Denmark (3.2 million)

	Incidence rate ratio (95% CI) of ICH in 2012–2018 vs 2005–2011				
	All of Denmark			Western Denmark only[1]	
	**Stroke Registry**	**Patient Registry**		**Stroke Registry**	**Patient Registry**
**Women[2]**					
**All ICH**					
Overall	0.88 (0.84; 0.91)	0.93 (0.89; 0.97)		0.86 (0.81; 0.91)	0.98 (0.93; 1.04)
20-64 years	0.92 (0.83; 1.02)	0.89 (0.82; 0.97)		0.99 (0.87; 1.12)	1.01 (0.90; 1.13)
65-74 years	0.76 (0.69; 0.84)	0.84 (0.77; 0.91)		0.78 (0.69; 0.88)	0.84 (0.75; 0.94)
75-84 years	0.89 (0.82; 0.96)	0.96 (0.89; 1.03)		0.83 (0.76; 0.92)	0.95 (0.87; 1.05)
≥85 years	0.93 (0.85; 1.02)	1.02 (0.94; 1.11)		0.88 (0.78; 0.99)	1.15 (1.03; 1.29)
**Fatal ICH**					
Overall	0.70 (0.65; 0.76)	0.85 (0.80; 0.91)		0.69 (0.62; 0.77)	0.90 (0.83; 0.98)
20-64 years	0.50 (0.39; 0.65)	0.67 (0.56; 0.80)		0.56 (0.42; 0.76)	0.68 (0.54; 0.86)
65-74 years	0.50 (0.41; 0.61)	0.68 (0.58; 0.79)		0.55 (0.43; 0.71)	0.70 (0.56; 0.86)
75-84 years	0.71 (0.62; 0.80)	0.89 (0.80; 0.99)		0.70 (0.60; 0.83)	0.91 (0.79; 1.05)
≥85 years	0.91 (0.80; 1.04)	1.03 (0.92; 1.15)		0.83 (0.69; 0.99)	1.16 (0.99; 1.36)
**Men[2]**					
**All ICH**					
Overall	0.87 (0.84; 0.91)	0.89 (0.86; 0.93)		0.84 (0.80; 0.89)	0.93 (0.88; 0.98)
20-64 years	0.92 (0.85; 0.99)	0.88 (0.83; 0.94)		0.90 (0.81; 0.99)	0.89 (0.81; 0.97)
65-74 years	0.86 (0.79; 0.93)	0.87 (0.80; 0.93)		0.81 (0.73; 0.89)	0.90 (0.82; 0.99)
75-84 years	0.84 (0.78; 0.91)	0.91 (0.85; 0.98)		0.80 (0.73; 0.89)	0.95 (0.86; 1.04)
≥85 years	0.87 (0.77; 0.99)	0.97 (0.87; 1.09)		0.88 (0.75; 1.04)	1.10 (0.95; 1.28)
**Fatal ICH**					
Overall	0.69 (0.64; 0.75)	0.74 (0.69; 0.79)		0.73 (0.66; 0.81)	0.82 (0.75; 0.90)
20-64 years	0.61 (0.50; 0.74)	0.61 (0.53; 0.70)		0.67 (0.53; 0.85)	0.68 (0.57; 0.83)
65-74 years	0.64 (0.55; 0.76)	0.68 (0.60; 0.78)		0.68 (0.56; 0.84)	0.73 (0.61; 0.87)
75-84 years	0.71 (0.62; 0.80)	0.78 (0.69; 0.87)		0.75 (0.64; 0.88)	0.86 (0.75; 1.00)
≥85 years	0.81 (0.67; 0.98)	0.97 (0.83; 1.14)		0.83 (0.65; 1.05)	1.09 (0.89; 1.34)
**Women & Men[3]**					
**All ICH**					
Overall	0.87 (0.85; 0.90)	0.91 (0.89; 0.94)		0.85 (0.82; 0.88)	0.95 (0.92; 0.99)
20-64 years	0.92 (0.86; 0.97)	0.89 (0.84; 0.93)		0.93 (0.86; 1.00)	0.93 (0.87; 1.00)
65-74 years	0.82 (0.77; 0.87)	0.85 (0.81; 0.90)		0.79 (0.73; 0.86)	0.87 (0.81; 0.94)
75-84 years	0.87 (0.82; 0.91)	0.94 (0.89; 0.98)		0.82 (0.76; 0.88)	0.95 (0.89; 1.02)
≥85 years	0.91 (0.85; 0.98)	1.01 (0.94; 1.08)		0.88 (0.80; 0.97)	1.13 (1.04; 1.24)
**Fatal ICH**					
Overall	0.70 (0.66; 0.74)	0.80 (0.76; 0.83)		0.71 (0.66; 0.76)	0.86 (0.81; 0.92)
20-64 years	0.57 (0.49; 0.66)	0.63 (0.56; 0.70)		0.63 (0.52; 0.76)	0.68 (0.59; 0.79)
65-74 years	0.58 (0.51; 0.66)	0.68 (0.62; 0.75)		0.63 (0.53; 0.73)	0.72 (0.63; 0.82)
75-84 years	0.71 (0.64; 0.77)	0.83 (0.77; 0.90)		0.73 (0.65; 0.82)	0.89 (0.80; 0.98)
≥85 years	0.88 (0.79; 0.98)	1.01 (0.92; 1.11)		0.83 (0.72; 0.96)	1.14 (1.00; 1.29)

[1]Eastern Denmark excluded as changes in administrative systems in 2016 (introduction of a new electronic health record) may have caused changes in completeness of registration of ICH in that part of Denmark.

Abbreviations: CI: confidence interval; ICH: intracerebral hemorrhage

[2]Age standardised (5-year bands) incidence rates used to calculate rate ratios

[3]Age (5-year bands) and sex standardised used to calculate rate ratios

© 2021 Hald SM et al. *JAMA Network Open.*

eTable 14. Incidence rate of verified spontaneous intracerebral hemorrhage per 100,000 person-years and prevalence of use of antithrombotic drugs among general population controls in Region of Southern Denmark, 2009-2017.

**Incidence of intracerebral hemorrhage**											
	**2009-2011**		**2012-2014**		**2015-2017**			**Incidence rate ratio (95% CI)**			
	**No. of cases[1]**	**sIR[2] (95% CI)**	**No. of cases[1]**	**sIR[2] (95% CI)**	**No. of cases[1]**	**sIR[2] (95% CI)**		**2012-2014 vs. 2009-2011**	***P*-value**	**2015-2017 vs. 2009-2011**	***P*-value**
**All ICH**											
All	892	34 (31; 36)	850	30 (28; 32)	967	32 (30; 35)		0.90 (0.82; 0.99)	0.8	0.97 (0.88; 1.06)	0.4
20-74 years	471	20 (18; 22)	430	17 (16; 19)	498	19 (17; 21)		0.87 (0.76; 0.99)	0.8	0.96 (0.84; 1.09)	0.4
≥75 years	421	156 (141; 171)	420	148 (134; 162)	469	153 (138; 168)		0.95 (0.83; 1.08)	0.4	0.98 (0.86; 1.12)	0.2
≥85 years	135	183 (153; 214)	175	221 (188; 256)	191	228 (194; 262)		1.21 (0.97; 1.51)	0.7	1.25 (1.00; 1.56)	0.8
**Fatal ICH[3]**											
All	338	13 (11-14)	321	11 (10;13)	344	11 (10; 13)		0.90 (0.78;1.03)	0.7	0.90 (0.78; 1.03)	0.7
20-74 years	139	5.8 (4.8;6.7)	118	4.7 (3.8;5.5)	133	4.9 (4.1;5.8)		0.81 (0.66;0.99)	0.9	0.86 (0.69;1.05)	0.4
≥75 years	199	74 (64;84)	203	71 (61;81)	211	69 (59;79)		0.96 (0.79; 1.16)	0.3	0.93 (0.78; 1.12)	0.7
≥85 years	72	98 (76-121)	91	115 (91;139)	107	129 (103;154)		1.17 (0.81; 1.68)	0.4	1.31 (0.78; 1.94)	0.7
**Use of antithrombotic drugs among general population controls[4,5]**											
		**2009-2011**		**2012-2014**		**2015-2017**		**Prevalence ratio (95% CI)**			
	**No. of controls**	**Percentage[2]**	**No. of controls**	**Percentage[2]**	**No. of controls**	**Percentage[2]**		**2012-2014 vs. 2009-2011**	***P*-value**	**2015-2017 vs. 2009-2011**	***P*-value**
**Any oral anticoagulant**											
All	1992	5.5	2422	7.5	3322	9.7		1.38 (1.30; 1.46)	<0.001	1.77 (1.68; 1.87)	<0.001
20-74 years	535	3.0	639	3.8	776	4.3		1.30 (1.16; 1.46)	<0.001	1.45 (1.30; 1.62)	<0.001
≥75 years	1457	8.0	1783	11	2546	15		1.41 (1.32; 1.51)	<0.001	1.91 (1.80; 2.03)	<0.001
≥85 years	416	6.9	660	10.8	1134	18		1.56 (1.38; 1.75)	<0.001	2.52 (2.27; 2.81)	<0.001
**VKA**											
All	1975	5.4	1926	6.0	1804 (5.4)	5.2		1.10 (1.04; 1.17)	0.002	0.97 (0.91; 1.03)	0.3
20-74 years	525	2.9	511	3.1	432 (2.6)	2.4		1.06 (0.94; 1.20)	0.344	0.82 (0.72; 0.94)	0.003
≥75 years	1450	7.9	1415	8.9	1372 (8.2)	8.2		1.13 (1.05; 1.21)	0.001	1.03 (0.96; 1.11)	0.3
≥85 years	415	6.9	505	8.3	566 (8.8)	8.7		1.20 (1.05; 1.35)	0.005	1.26 (1.12; 1.43)	<0.001
**DOAC**											
All	18	0.0	518	1.6	1548 (4.6)	4.5		33 (21; 52)	<0.001	92 (58; 146	<0.001

© 2021 Hald SM et al. *JAMA Network Open.*

20-74 years	10	0.1	135	0.8	349 (2.1)	1.9		15 (7.8; 28)	<0.001	35 (19; 66)	<0.001
≥75 years	8	0.0	383	2.4	1199 (7.2)	7.2		56 (28; 112)	<0.001	166 (83; 333)	<0.001
≥85 years	<5	0	161	2.6	578 (9.0)	8.9		81 (20; 326)	<0.001	275 (69; 1101)	<0.001
**Any antiplatelet drug**											
All	9840	27	8483	26	7664	22		0.98 (0.95; 1.00)	0.082	0.83 (0.81; 0.85)	<0.001
20-74 years	3020	17	2657	16	2440	14		0.97 (0.93; 1.02)	0.227	0.83 (0.79; 0.88)	<0.001
≥75 years	6820	37	5826	37	5224	31		0.99 (0.96; 1.01)	0.304	0.84 (0.81; 0.86)	<0.001
≥85 years	2465	41	2430	40	2238	35		0.97 (0.93; 1.01)	0.188	0.84 (0.80; 0.88)	<0.001

Abbreviations: DOAC: direct oral anticoagulant; ICH: intracerebral hemorrhage; sIR: standardized incidence rate; VKA: vitamin K antagonist

[1]Cases verified based on medical record information including brain imaging reports. In 188 cases (6%), where medical records could not be traced, we used the results of 100 imputed data sets with imputation based on published positive predictive values for the registries used for case-identification (see eMethods).

[2]Age- and sex-standardized to 2011 population in Denmark.

[3]Died within 30 days of ICH onset. In 74 (7.3%), where medical records could not be traced, we used imputed data as described under footnote 1 above.

[4]Controls were randomly selected among individuals in the general population that matched ICH-cases with regard to age, sex, and calendar time (index date) and resided in Region of Southern Denmark.

[5]Current use defined as treatment episode with the drug within a month (0-30 days) of the date of selection as control.

© 2021 Hald SM et al. *JAMA Network Open.*

eTable 15. Percentage of cases and their general population controls classified as current users of antithrombotic drugs, Denmark 2005-2018

AGED 20-64 years															
	CASES								CONTROLS						
	Any AT	Any AP	Any OAC	ASA	Clopi	DOAC	VKA		Any AT	Any AP	Any OAC	ASA	Clopi	DOAC	VKA
2005	19.5	16.8	3.7	16.2	1.7	0.0	3.7		8.7	7.8	1.1	7.7	0.5	0.0	1.1
2006	21.4	16.8	5.8	15.9	.	0.0	5.8		8.9	8.1	1.0	8.0	0.4	0.0	1.0
2007	24.5	20.9	6.3	19.7	.	0.0	6.3		10.4	9.3	1.5	9.0	0.7	0.0	1.5
2008	21.7	16.7	7.0	16.7	.	0.0	7.0		10.3	9.1	1.5	8.9	0.7	0.0	1.5
2009	22.9	18.9	6.3	18.3	1.4	0.0	6.3		9.8	8.8	1.3	8.6	0.7	0.0	1.3
2010	19.4	16.6	4.5	15.3	2.9	0.0	4.5		9.7	8.6	1.4	8.3	0.8	*	1.4
2011	22.6	18.2	6.1	16.9	3.2	0.0	6.1		9.9	8.9	1.3	8.4	0.9	*	1.3
2012	21.4	17.7	4.7	15.4	3.7	0.0	4.7		10.2	9.0	1.4	8.3	1.1	0.2	1.2
2013	18.2	15.8	3.7	12.5	4.0	*	3.0		9.2	8.1	1.4	7.2	1.2	0.2	1.1
2014	18.4	14.8	4.2	10.6	5.3	*	3.2		8.8	7.6	1.4	6.5	1.5	0.3	1.1
2015	17.7	13.7	4.7	11.0	3.7	*	4.0		8.8	7.4	1.6	6.2	1.5	0.6	1.0
2016	17.4	14.7	3.9	8.1	7.5	1.8	2.4		8.5	7.1	1.6	5.7	1.7	0.6	1.0
2017	14.4	7.7	6.6	4.8	3.0	3.0	3.7		8.0	6.6	1.5	5.0	1.8	0.8	0.7
2018	19.8	15.3	6.5	8.4	7.3	5.0	*		8.5	6.8	1.9	5.2	1.9	1.0	0.9
AGED 65-74 YEARS															
	CASES								CONTROLS						
	Any AT	Any AP	Any OAC	ASA	Clopi	DOAC	VKA		Any AT	Any AP	Any OAC	ASA	Clopi	DOAC	VKA
2005	39.9	33.7	9.6	33.3	1.7	0.0	9.6		24.6	21.9	3.7	21.5	1.0	0.0	3.7
2006	47.1	38.4	12.0	36.6	2.5	0.0	12.0		26.7	23.8	4.2	23.4	1.3	0.0	4.2
2007	45.8	37.2	13.2	35.8	3.5	0.0	13.2		27.6	24.2	4.9	23.6	1.4	0.0	4.9
2008	51.0	40.9	15.4	40.5	2.3	0.0	15.4		27.2	24.1	4.4	23.5	1.3	0.0	4.4
2009	43.7	35.7	12.6	34.5	2.2	0.0	12.6		28.4	25.1	4.6	24.4	1.6	*	4.6
2010	46.8	38.5	16.1	36.5	2.0	0.0	16.1		28.4	25.3	4.4	24.4	1.8	0.1	4.4
2011	47.1	40.1	12.4	36.1	5.5	*	12.0		27.9	24.5	4.8	23.3	2.2	0.1	4.7
2012	49.1	41.3	13.0	34.9	10.0	*	12.6		29.7	25.7	5.4	23.8	2.8	0.7	4.8
2013	42.0	31.9	13.2	28.1	6.9	1.6	11.7		28.1	23.7	5.6	21.3	3.2	1.2	4.5
2014	44.7	37.3	11.2	27.1	11.5	2.7	8.5		25.6	20.8	5.8	17.8	3.7	1.7	4.2
2015	49.5	35.3	17.5	27.1	10.9	4.6	12.9		26.8	21.1	6.4	17.4	4.4	2.6	3.9
2016	46.4	28.7	19.8	20.6	11.3	9.1	10.7		26.9	20.4	7.4	15.9	5.2	3.2	4.3
2017	45.3	30.7	17.1	20.3	12.7	9.2	8.2		27.2	20.4	7.3	15.9	5.3	3.9	3.6
2018	40.3	27.0	14.0	15.0	13.3	7.2	7.2		26.5	19.3	7.7	14.3	5.8	4.9	2.8
AGED 75+ YEARS															

© 2021 Hald SM et al. *JAMA Network Open.*

	CASES								CONTROLS						
	**Any AT**	**Any AP**	**Any OAC**	**ASA**	**Clopi**	**DOAC**	**VKA**		**Any AT**	**Any AP**	**Any OAC**	**ASA**	**Clopi**	**DOAC**	**VKA**
2005	48.9	41.0	11.5	40.4	1.4	0.0	11.5		37.3	33.5	5.0	33.0	1.3	0.0	5.0
2006	49.3	41.2	13.5	40.0	2.2	0.0	13.5		39.5	35.0	6.0	34.3	1.5	0.0	6.0
2007	54.4	44.9	14.9	43.7	1.7	0.0	14.9		40.6	35.6	6.6	34.9	1.6	0.0	6.6
2008	55.3	45.4	16.8	44.0	2.4	0.0	16.8		42.8	37.5	7.2	36.5	1.8	0.0	7.2
2009	59.1	45.5	18.9	44.7	1.7	*	18.8		42.8	37.5	7.3	36.5	2.0	0.0	7.3
2010	60.5	49.3	18.3	47.1	4.4	0.0	18.3		44.3	38.8	7.6	37.5	2.4	0.0	7.6
2011	58.2	45.8	18.1	43.5	4.4	0.0	18.1		44.2	38.4	8.2	36.3	3.3	0.1	8.1
2012	58.3	45.0	20.8	39.1	8.2	1.5	19.4		45.5	38.5	9.5	35.4	4.6	1.0	8.6
2013	57.5	42.1	21.4	35.6	8.7	2.9	18.8		44.9	36.5	10.6	32.5	5.6	2.1	8.6
2014	58.6	41.9	23.0	34.2	10.3	4.4	18.6		44.9	34.7	12.0	29.2	6.8	3.5	8.6
2015	61.4	40.4	24.5	28.7	14.4	8.2	16.4		44.3	32.3	13.8	26.0	7.4	5.5	8.4
2016	58.5	35.2	25.3	20.3	16.6	10.3	15.6		44.6	31.5	14.4	24.0	8.6	6.6	8.0
2017	56.3	31.4	28.3	21.7	11.7	13.9	14.6		44.7	29.8	16.1	21.6	9.2	8.7	7.5
2018	58.8	31.2	29.8	19.1	14.2	19.4	10.5		44.8	29.0	16.9	20.4	9.5	10.7	6.5
**ALL**															
	**CASES**								**CONTROLS**						
	**Any AT**	**Any AP**	**Any OAC**	**ASA**	**Clopi**	**DOAC**	**VKA**		**Any AT**	**Any AP**	**Any OAC**	**ASA**	**Clopi**	**DOAC**	**VKA**
2005	39.7	33.5	9.1	32.9	1.5	0.0	9.1		27.6	24.7	3.8	24.3	1.0	0.0	3.8
2006	41.2	33.9	11.1	32.7	2.0	0.0	11.1		28.4	25.3	4.3	24.8	1.2	0.0	4.3
2007	44.0	36.3	12.1	35.1	2.0	0.0	12.1		29.2	25.6	4.8	25.1	1.3	0.0	4.8
2008	45.5	36.8	13.9	36.1	1.9	0.0	13.9		30.9	27.2	5.1	26.5	1.4	0.0	5.1
2009	45.3	35.7	13.9	34.8	1.7	*	13.8		30.2	26.6	5.0	25.9	1.5	0.0	5.0
2010	46.7	38.3	14.2	36.4	3.4	0.0	14.2		31.7	27.9	5.3	27.0	1.8	0.0	5.2
2011	45.9	37.0	13.5	34.5	4.3	*	13.4		31.2	27.2	5.6	25.8	2.4	0.1	5.5
2012	46.2	36.8	14.6	31.7	7.4	0.8	13.8		32.4	27.6	6.4	25.5	3.2	0.7	5.8
2013	43.7	32.9	14.9	27.9	7.1	2.0	13.0		31.7	26.2	7.0	23.4	3.9	1.4	5.7
2014	44.8	33.9	15.2	26.4	9.4	3.1	12.0		30.9	24.4	7.8	20.6	4.7	2.3	5.6
2015	47.5	32.5	17.8	23.9	10.9	5.4	12.4		31.2	23.3	8.9	19.0	5.2	3.5	5.5
2016	44.4	28.0	18.2	17.3	12.7	7.7	10.8		30.3	22.1	9.2	17.0	5.9	4.1	5.1
2017	44.6	26.2	20.9	17.7	10.0	10.4	10.7		32.6	22.6	10.8	16.7	6.7	5.8	5.1
2018	44.9	26.4	20.2	15.5	12.3	12.9	7.5		31.7	21.4	11.1	15.3	6.8	7.0	4.2

Abbreviations: AP: antiplatelet; ASA: low-dose aspirin; AT: antithrombotic; Clopi: Clopidogrel; OAC: oral anticoagulant; VKA: vitamin K antagonist
Note: 0.0 indicates no current users; * indicates percentages that were not calculated due to sparse data (< 5 current users)

© 2021 Hald SM et al. *JAMA Network Open.*

eFigure 1 Incidence rate of intracerebral hemorrhage in Denmark by source used to identify cases. Denmark, 2005-2018

Danish Stroke Registry

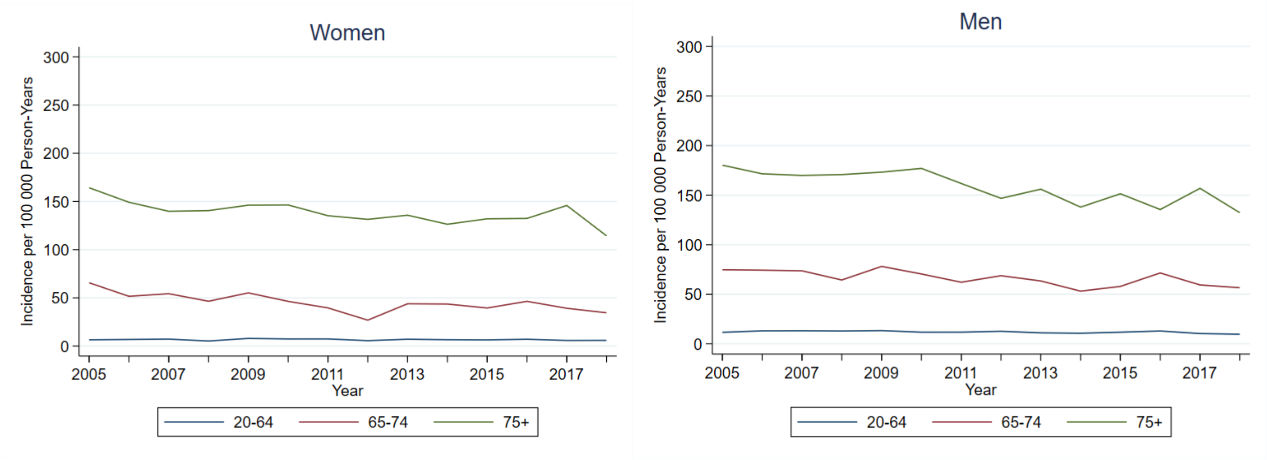

Danish National Patient Registry (source for main analysis)

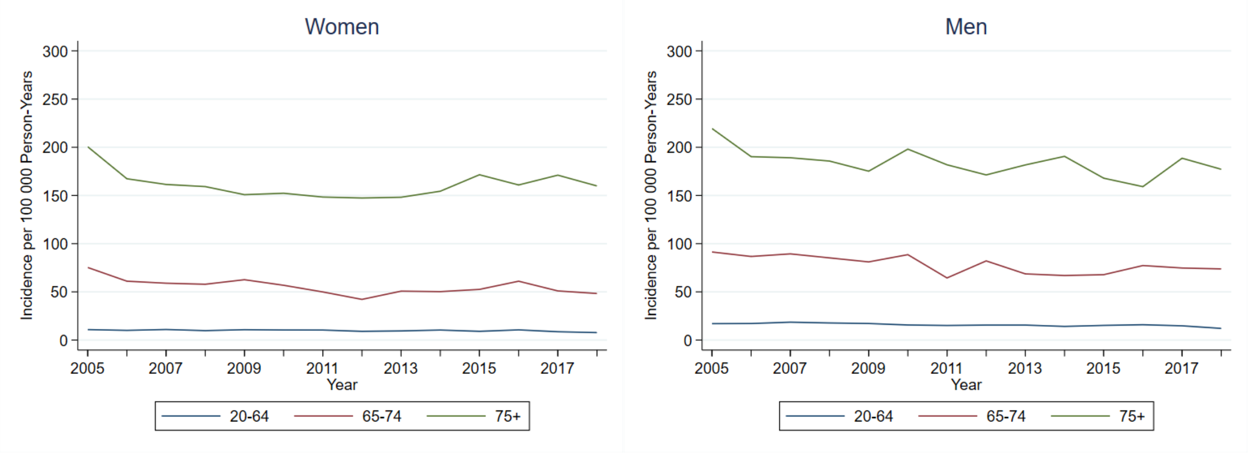

© 2021 Hald SM et al. *JAMA Network Open.*

eFigure 2 Incidence rate of intracerebral hemorrhage by source used to identify cases. Western Denmark, 2005-2018

Danish Stroke Registry

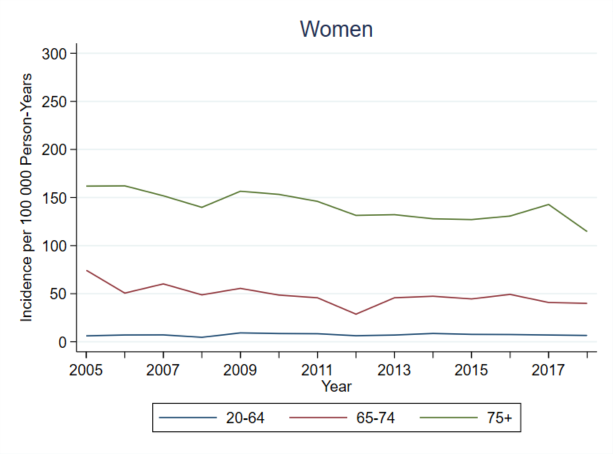

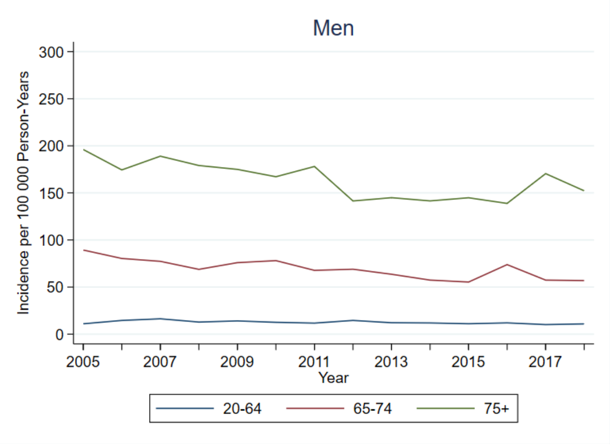

Danish National Patient Registry (source main analyses)

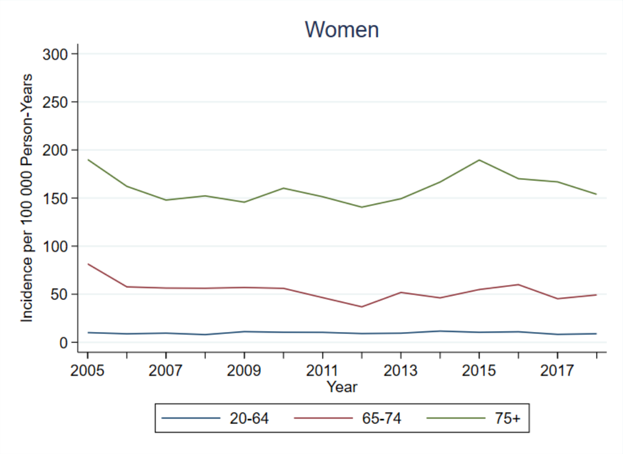

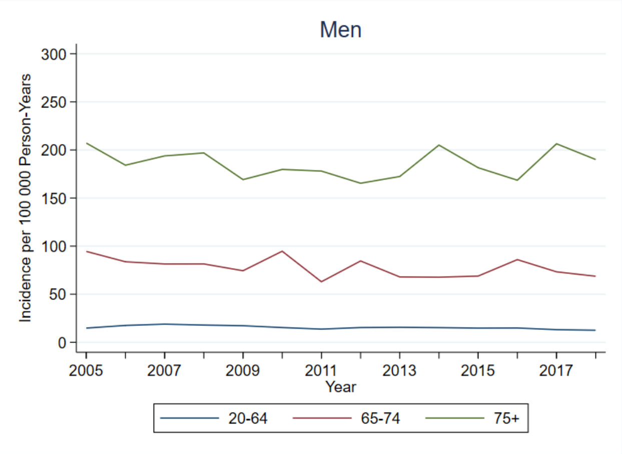

© 2021 Hald SM et al. *JAMA Network Open.*

eFigure 3 Standardized incidence rates of verified spontaneous intracerebral hemorrhage (ICH) and prevalence of antithrombotic drug use in general population controls in Region of Southern Denmark (RSD), 2009-2017.
Also included, nationwide standardized incidence rates of ICH based on Stroke Registry and Patient Registry.

A. Standardized incidence rates of verified spontaneous ICH in RSD and of ICH based on nationwide (DK) registries

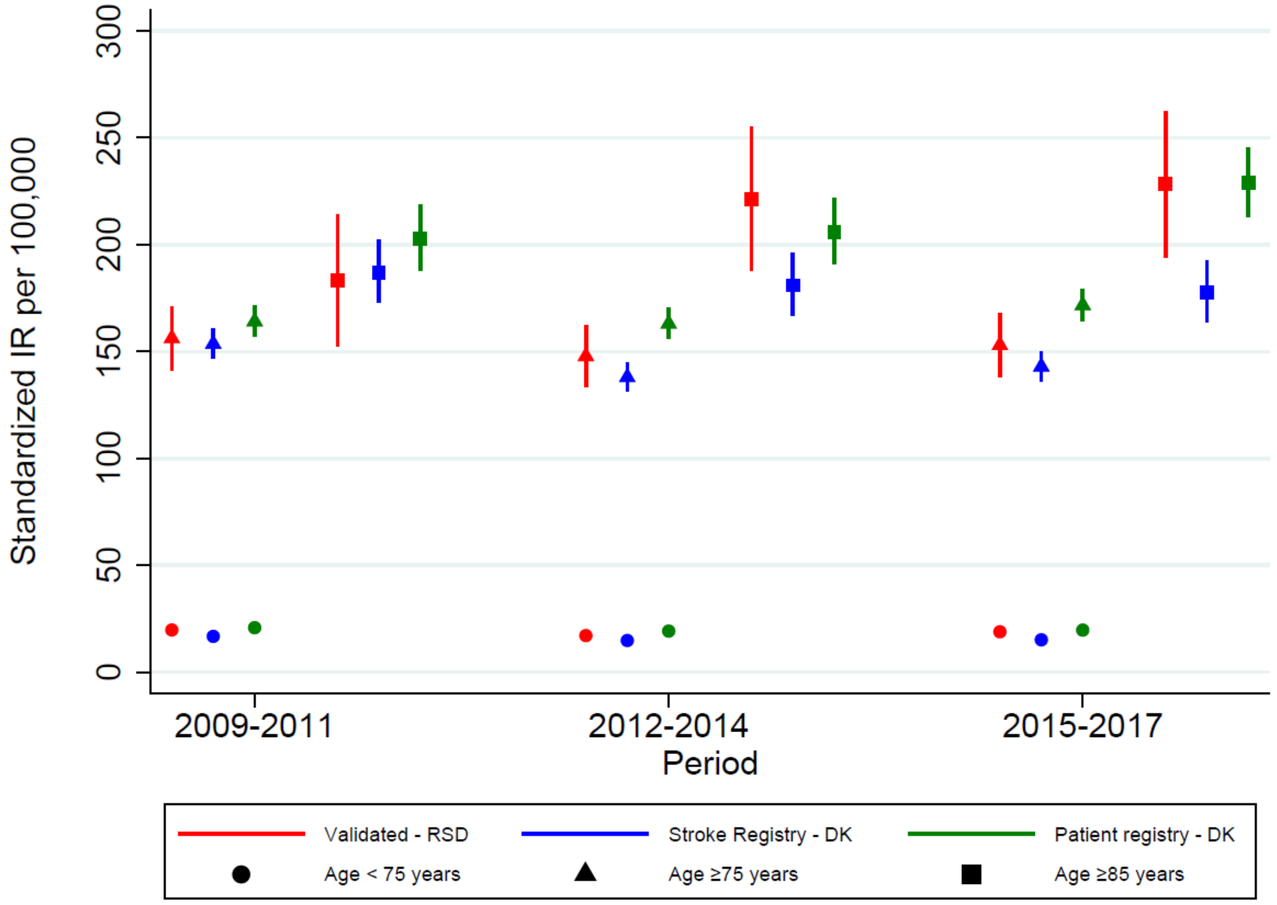

© 2021 Hald SM et al. *JAMA Network Open.*

B. Prevalence of anticoagulant use in general population controls, RSD 2009-2017

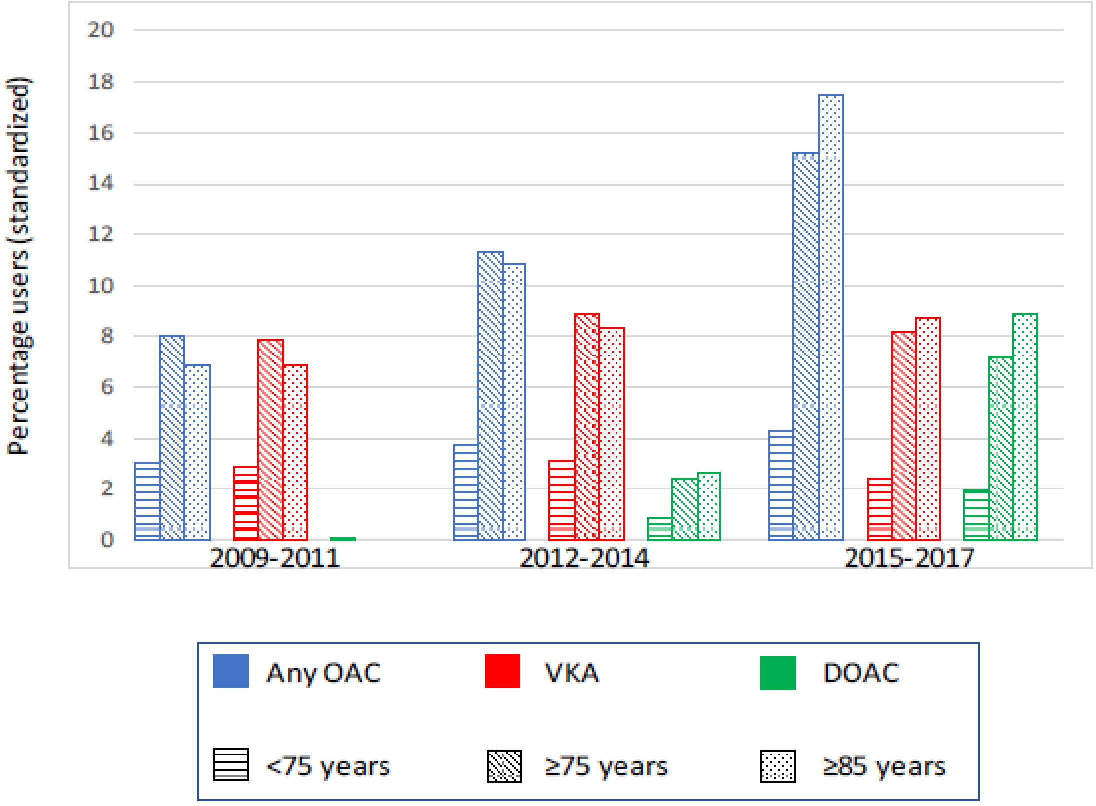

C. Prevalence of antiplatelet use in general population controls, RSD 2009-2017

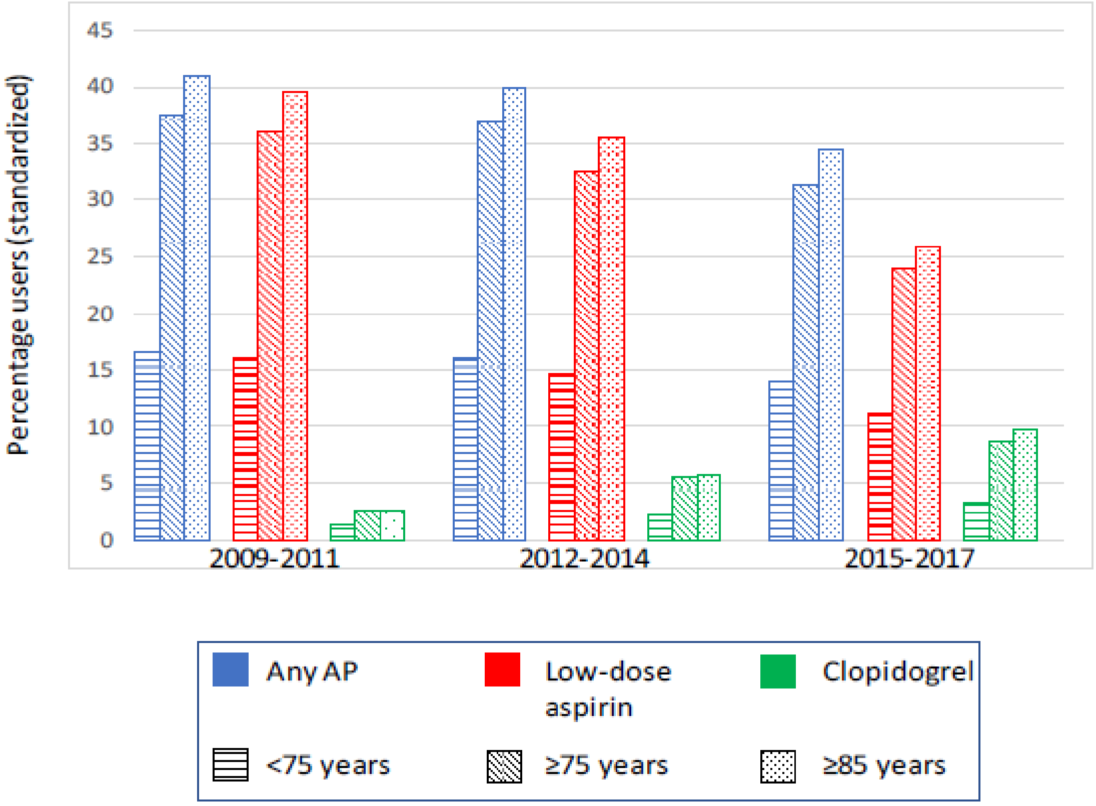

© 2021 Hald SM et al. *JAMA Network Open.*

eFigure 4. Annual percentage of current use of antithrombotic drugs among cases with intracerebral hemorrhage and their general population controls, Denmark 2005-2018

Antiplatelet drugs

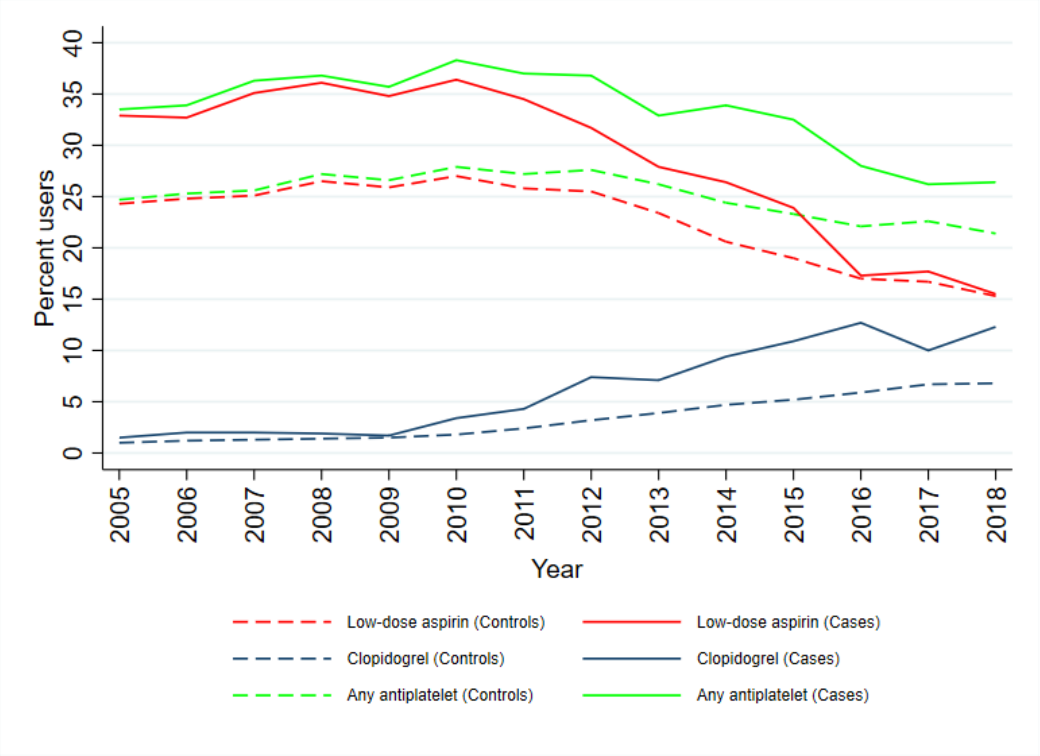

Oral anticoagulant drugs

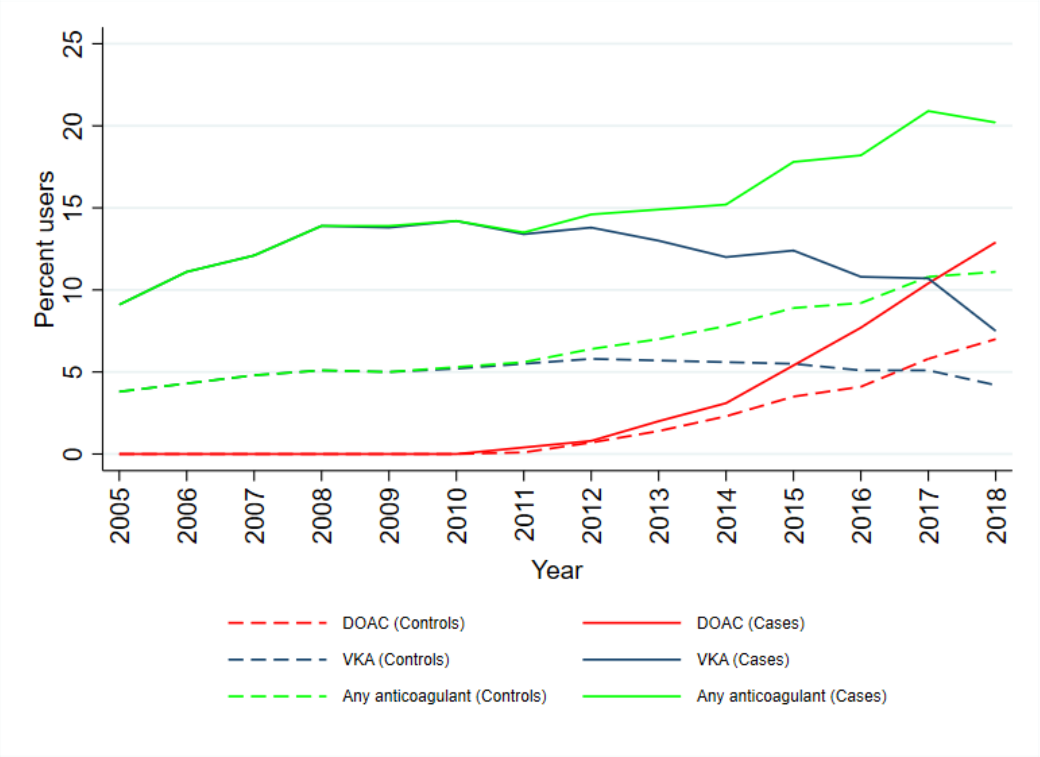

© 2021 Hald SM et al. *JAMA Network Open.*
